# Maximizing human effort for analyzing scientific images: A case study using digitized herbarium sheets

**DOI:** 10.1002/aps3.11370

**Published:** 2020-07-01

**Authors:** Laura Brenskelle, Rob P. Guralnick, Michael Denslow, Brian J. Stucky

**Affiliations:** ^1^ Florida Museum of Natural History University of Florida Gainesville Florida USA; ^2^ Department of Biology University of Florida Gainesville Florida USA

**Keywords:** citizen science, herbarium specimens, image annotation, machine learning, phenology, specimen images

## Abstract

**Premise:**

Digitization and imaging of herbarium specimens provides essential historical phenotypic and phenological information about plants. However, the full use of these resources requires high‐quality human annotations for downstream use. Here we provide guidance on the design and implementation of image annotation projects for botanical research.

**Methods and Results:**

We used a novel gold‐standard data set to test the accuracy of human phenological annotations of herbarium specimen images in two settings: structured, in‐person sessions and an online, community‐science platform. We examined how different factors influenced annotation accuracy and found that botanical expertise, academic career level, and time spent on annotations had little effect on accuracy. Rather, key factors included traits and taxa being scored, the annotation setting, and the individual scorer. In‐person annotations were significantly more accurate than online annotations, but both generated relatively high‐quality outputs. Gathering multiple, independent annotations for each image improved overall accuracy.

**Conclusions:**

Our results provide a best‐practices basis for using human effort to annotate images of plants. We show that scalable community science mechanisms can produce high‐quality data, but care must be taken to choose tractable taxa and phenophases and to provide informative training material.

Efforts to digitize herbarium specimens have expanded past transcribing textual content on labels and now often include broad‐scale specimen imaging. With the burgeoning growth of imaged historical plant specimen data and associated metadata, these data are poised to provide a crucial data resource about phenotypic and phenological traits (e.g., Yost et al., 2018; Lorieul et al., 2019; Park et al., 2019), extending over centuries of plant collecting effort. As of March 2020, iDigBio (Page et al., 2015; http://www.idigbio.org) alone provides 19 million imaged plant specimens. Despite the promise of these image resources, generating usable trait annotations from specimen images remains challenging, especially as the scale of data resources continues to grow. Building such data sets by hand is incredibly time consuming and presumably requires at least some expert knowledge to assess the taxa of interest for a given trait. Such data sets are critical for enabling scientific research and for training machine learning algorithms to increase capacity.

Little guidance is available for how to best leverage human effort to generate image annotations. Recent work has tested the performance of for‐credit students versus paid crowdsourcing approaches (Zhou et al., 2018). Other methods range from individual researchers annotating a case study taxon with which they are familiar, to local volunteer efforts within an institution or organization, to citizen or community science approaches.

We provide a case study investigating the quality of human image annotations using two methods: annotation by in‐person volunteers, and annotation by community scientists on the Notes from Nature platform (Hill et al., 2012). We focused on a relatively simple set of phenological annotations: presence or absence of flowers, fruits, and unfolded leaves. Although detecting traits such as leaves, flowers, and fruits sounds simple enough, we show that it is not trivial even in best‐case scenarios, and rarely do imaged herbarium sheets reach that best‐case threshold (e.g., Fig. 1). The genera *Prunus* L. and *Acer* L. were specifically selected because of the abundance of records in iDigBio, because they are commonly included in phenology monitoring programs such as the USA National Phenology Network (https://www.usanpn.org/), and because their flowering timing is recognized as one of the signifiers of the onset of spring (Li and Liu, 2011).

**FIGURE 1:**
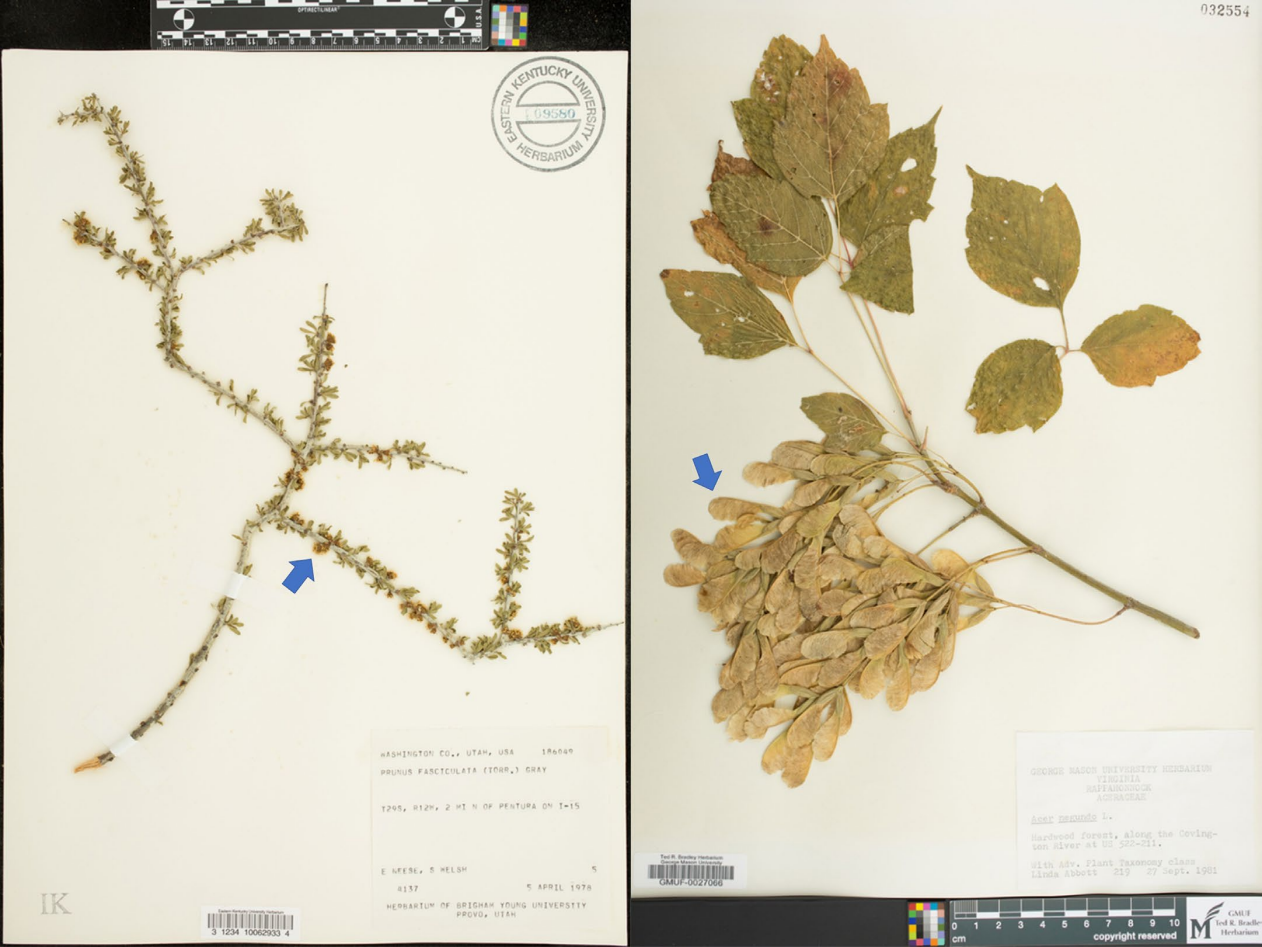
Examples of *Prunus* (left) and *Acer* (right) specimens included in our studies. The left image from the Ronald L. Jones Herbarium at Eastern Kentucky University (https://www.idigbio.org/portal/mediarecords/f45a2afe‐38b5‐414a‐9ab3‐85f30ae816d9) shows an example of a *Prunus fasciculata* (Torr.) A. Gray specimen. This image shows unfolded leaves present, fruits absent, and flowers present. The flowers on this species are inconspicuous and best seen by zooming into the full‐scale image, but we have indicated where flowers can be seen by the blue arrow on the image. The image on the right is an *Acer negundo* L. specimen from the Ted R. Bradley Herbarium at George Mason University (https://www.idigbio.org/portal/records/05320ea2‐d731‐4878‐a308‐f75bcf45e590). This image shows unfolded leaves present, fruits present (indicated by the blue arrow), and flowers absent.

We focused on six main questions:

1. Are certain phenological traits or taxonomic groups easier to annotate than others?
2. How do differences among volunteers affect annotation accuracy?
3. Does speed of annotation matter for quality; e.g., are slower annotators more accurate?
4. Do annotators improve with practice or fatigue with more effort?
5. Is there a difference in annotation accuracy for Notes from Nature versus in‐person volunteers?
6. Does majority‐rule scoring increase accuracy?

We discuss, in particular, the significance of the subject matter expertise of volunteers, time taken to complete tasks, and individual volunteer ability. We also provide best‐practices suggestions for researchers who wish to use human effort to generate accurate image annotations.

## METHODS

### Image gathering and filtering

The image sets used for this project were random samples of 3000 herbarium sheet images from iDigBio for each of two plant genera, *Acer* and *Prunus*. To generate these image sets, we used the iDigBio data portal (https://www.idigbio.org/portal/search) in July (*Acer*) and August (*Prunus*) of 2018 to download all iDigBio records identified to the target genera that included image data. We then downloaded all images for each iDigBio record and, to avoid the possibility of a single herbarium sheet being included more than once in the final image sets, we flagged all herbarium specimens (as identified by iDigBio’s *coreid* value) that had multiple images. Next, we randomly sampled 3000 images for each genus, ensuring that no specimen was represented by more than one image and only using images with a minimum dimension of at least 1024 pixels. We manually inspected all images in each sample and removed all images that were not of herbarium sheets, images of specimens that did not actually belong to the target genera, and images that were of such poor quality as to be virtually uninterpretable (e.g., images with incorrect color values, severe focus problems, etc.). Any such images were replaced by random selection from the remaining pool of images, thus keeping the total sample sizes at 3000 images for each genus.

### Generating a “gold‐standard” set of annotated images

Testing the accuracy of various image annotation methods requires a “gold‐standard” set of image annotations that are of very high accuracy (as close to perfect as reasonably achievable). We used the random samples of 3000 images for each target plant genus to develop a set of gold‐standard image annotations focused on three phenological traits assessed for each specimen as a whole: whether unfolded leaves are present or absent, whether flowers are present or absent, and whether fruits are present or absent. Precise definitions for these phenological traits were taken from the Plant Phenology Ontology (Stucky et al., 2018). Our procedure for generating the gold‐standard data set consisted of first annotating all images as a cooperative effort among three co‐authors (L.B., B.J.S., R.P.G.), then using a process of iterative refinement based on integrating outputs from other image annotation techniques.

We began with all three co‐authors working together to generate annotations for all three phenological traits for all 6000 images. The 3000 images for each genus were divided evenly among the co‐authors (i.e., 1000 images per author), and each co‐author worked independently to score each trait on their subset of images. During this first pass through the images, we flagged any images for which the correct annotations were ambiguous or not immediately obvious. We then reconvened for discussion and consensus scoring of these flagged images, culminating in a precise, consensus protocol for scoring each phenological trait for each genus. To document the final annotation protocols in detail, we developed a phenology trait handbook for each genus, illustrated with herbarium specimen images and botanical drawings, that provides examples of all phenological traits, including examples and discussion of especially difficult specimens and species (Appendices S1, S2). These handbooks were further developed for use in our volunteer phenology annotation experiments, described below.

After generating the initial gold‐standard set of annotations for all 6000 herbarium specimen images, we further refined the data set by incorporating the outputs of three alternative image labeling techniques: classifications from convolutional neural networks (CNNs), an in‐person volunteer annotation experiment (described below), and an online annotation experiment (also described below). For each of these, we examined all cases where the results of the alternative image annotation technique disagreed with the gold‐standard image annotations. All three co‐authors met to jointly examine each of these cases, and we updated the gold‐standard set of image annotations as needed. For classifications with CNNs, we used subsets of our initial gold‐standard data set, augmented by affine image transformations (Perez and Wang, 2017), to train CNNs based on the VGG16 (Simonyan and Zisserman, 2014) and DenseNet (Huang et al., 2017) architectures, and we used the trained CNNs to annotate all remaining images not in the training subsets. This work will be described in more detail in a future contribution. Accuracy measurements for all volunteer image annotations were determined via comparison with the gold‐standard data set.

### In‐person volunteer annotation

For our first volunteer image annotation experiment, we recruited 18 local affiliates, students, and staff at the Florida Museum of Natural History. Prior to annotating any images for the study, we asked these volunteers to fill out a brief survey indicating their academic career level and previous botanical experience (Appendix S3). All work with in‐person volunteers, including survey‐based data collection, was approved by the University of Florida Institutional Review Board (#IRB201802451), and we obtained written consent from all volunteers prior to their participation.

Each volunteer was assigned a target genus, with half the volunteers assigned to *Acer* and half to *Prunus*, and each volunteer was asked to score a random sample of 200 images of their target genus for each trait (flowers, fruits, and unfolded leaves). The random samples of 200 images were drawn from the larger sets of 3000 images described above, and a different sample (drawn with replacement) was used for each trait. Each volunteer was provided with brief (~15 minutes), in‐person training to explain the image annotation task, describe the phenological traits of interest, and learn to use the annotation software. Training was delivered by either L.B. or B.J.S. (or both). In addition, volunteers were provided with print and digital copies of the phenology annotation handbook for their target genus, which included example images and written explanations for the three phenological traits to be scored (Appendices S1, S2). Aside from this, volunteers were not allowed to receive any help with annotation decisions, but they were allowed to ask general questions about the meanings of the phenological terminology or about how to use the software. We also allowed the volunteers to choose the order in which they scored the phenological traits.

The volunteers used an early version of ImageAnt (https://gitlab.com/stuckyb/imageant) for all image annotation tasks. ImageAnt is a software tool developed by B.J.S. to facilitate efficient image annotation that provides a graphical interface for viewing the images and annotating the phenological traits. Users were able to freely zoom in or out on any portion of the image up to the maximum resolution available in the original source image.

### Notes from Nature annotation expeditions

For our second volunteer image annotation experiment, we used Notes from Nature (NfN; Hill et al., 2012), an online platform for community science image annotations. NfN is a community science platform that connects natural history collections data providers with volunteers who can help with tasks related to transcription and annotation of specimen images. The recent versions of NfN utilize an “expedition” model, in which sets of images are bundled into a theme. That model has successfully led to over 200 completed expeditions.

We created two NfN expeditions, one for each genus. Each of these expeditions contained 3000 images, but it was later found that there were two images in the *Prunus* data set that were not actually specimens of *Prunus*. These images were subsequently removed, making the *Prunus* data set slightly less than 3000 images. The expeditions were configured to require that each image received three independent annotations for each trait from three different volunteers. We used these triplicate image annotations to test the accuracy of consensus (majority rule; two out of three) image annotations in comparison to single annotations. NfN volunteers were provided with the same genus‐specific help guides used in the in‐person volunteer study, reformatted for presentation on the NfN platform. NfN volunteers received no additional training. However, L.B. and R.P.G. did use the NfN Talk feature to engage with online volunteers who had questions about phenological definitions.

NfN supports image files that are 1 MB in size or less for quicker display and more efficient storage, and many of the original images from iDigBio had to be reduced in size for use with NfN. NfN requires images in JPEG format, which uses a lossy image compression scheme. In general, image files that use lossy compression can be reduced in size in two main ways: either by downsampling the image (i.e., reducing the pixel count) or by increasing the compression ratio. We used a combination of both strategies to produce images for NfN that retained as much detail as possible. Our general approach was to use more aggressive image compression first, only downsampling images when we could not achieve the required image file size and quality through compression alone. Specifically, the JPEG compression scheme uses a “quality” parameter, which ranges from 0–100, to control the output compression ratio. A quality value of 100 produces the highest‐fidelity output images, and decreasing quality values result in decreasing output file size along with decreasing image quality. By visually inspecting a sample of iDigBio images that we subjected to a range of JPEG compression quality values, we determined that quality values of 60 and above resulted in images that retained sufficient detail for phenological analysis. For each image, then, we used the following algorithm to obtain an output image of the required size.

1. If the image file size is ≤1 MB, stop.
2. If the JPEG compression quality value is >60, decrease the quality value by 5, recompress the image, and go to 1. Otherwise, go to 3.
3. Scale the image so the dimensions are reduced by 5%. Go to 1.

The NfN expedition for *Prunus* was launched on 6 February 2019 and was completed by NfN volunteers on 14 June 2019. The expedition for *Acer* was launched on 18 June 2019 and was completed on 21 July 2019.

### Data analyses of the in‐person study

For the in‐person volunteer study, we tested the effects of phenological trait, genus, volunteer, previous botanical experience, academic career level, and total annotation time (the total amount of time the volunteer spent classifying all 600 assigned images) on annotation accuracy. For each record, we determined if a volunteer’s annotation was correct in comparison to the gold‐standard data set, and gave it an accuracy score of 0 or 1 (incorrect or correct). This accuracy score was used as a binary response variable in a generalized linear mixed model (GLMM) with the logit link function; the predictor variables *trait*, *genus*, *total_time*, *position* (academic career level of the volunteer), and *prior_experience* as fixed effects; and *volunteer* as a random intercept effect. We used the R statistical package (R Core Team, 2019) for analyses, and more specifically the glmer() function of the lme4 package (Bates et al., 2015). The glmer() function by default uses a combination of the Nelder‐Mead and BOBYQA optimizers (Powell, 2009; S. G. Johnson, 2014). For this and all subsequent analyses, we used the default combination of optimizers in all cases except when the model had two random effects, in which case we used only Nelder‐Mead.

In order to assess the importance of continuous predictors (e.g., *total_time*), we examined predictor *P* values from model summaries. We tested categorical predictor importance by dropping each categorical predictor from the model one at a time, fitting the reduced models, and determining via log‐likelihood tests if the reduced models were significantly different from models that included the dropped predictor. We also report Akaike information criterion (AIC) as ΔAIC, which shows the difference in the AIC of the “best” model in comparison to the full model (Tables 1, 2). To assess random effects, we used the same model comparison technique as for fixed effects and Nakagawa’s *R* ^2^ (Nakagawa and Schielzeth, 2013; P. C. D. Johnson 2014; Nakagawa et al., 2017).

**Table 1:** Best‐fit models for the individual studies and the combined model comparing between the two studies.

Study	Best model parameters	Nonsignificant parameters	ΔAIC^a^	*n*	Marginal *R* ^2^	Conditional *R* ^2^
In‐person volunteer	Fixed effects: genus + trait + genus*trait Random effects: volunteer	Fixed effects: prior experience, career position, total time	2.3	10,795	0.016	0.062
Notes from Nature	Fixed effects: genus + trait + image count Random effects: volunteer	Fixed effects: time, genus*trait	1	47,535	0.079	0.274
Combined	Fixed effects: genus + trait + study Random effect: volunteer	Fixed effects: genus*trait	~0	58,330	0.026	0.211
Majority rule	Fixed effects: method + genus + trait	—	—	17,984	0.009	—

*Note: n* = number of trait annotations included in a data set.

^a^ Δ AIC shows the difference in Akaike information criterion of the “best” model in comparison to the full model. Where no Δ AIC value is reported, the full model was the best.

**Table 2:** Best‐fit models testing whether accuracy in the two studies varied by species. For these models, we filtered out species with fewer than 30 images in the data set. In both the in‐person and Notes from Nature annotations, *species* is significant in the models.

Study	Best model parameters	ΔAIC^a^	*n*	Marginal *R* ^2^	Conditional *R* ^2^
In‐person volunteer	Fixed effects: genus + trait + prior experience Random effects: volunteer, species	4.1	9044	0.004	0.104
Notes from Nature	Fixed effects: genus + trait + image count Random effects: volunteer, species	2	44,754	0.076	0.301

*Note: n* = number of trait annotations included in a data set.

^a^ ΔAIC shows the difference in Akaike information criterion of the “best” model in comparison to the full model. Where no ΔAIC value is reported, the full model was the best.

### Data analyses of the Notes from Nature study

Because there was a large disparity among the number of images annotated by individual volunteers, we set a cut‐off criterion that only volunteers who annotated 30 or more images would be used for the statistical analyses. We again determined an accuracy score for the remaining phenological annotations by comparing them to the gold‐standard data set. We fit a GLMM using the accuracy score as the binary response variable, logit link function, and *genus*, *trait*, *time* (the amount of time the volunteer spent on the image), and *image_count* (the total number of images a given volunteer classified) as fixed effects, and *volunteer* as a random intercept effect. We found the best‐fit model by repeating the same method as described above for the volunteer data set.

Additionally, for the NfN study we examined whether accuracy improved with majority‐rule consensus annotations (two out of three annotations agreeing) in comparison to the accuracy of single scorers per image. We did this by first eliminating images lacking triplicate annotations (a bug in the NfN platform causes a small number of images to not receive the required number of independent annotations). Then, we generated a majority‐rule classification for each of the remaining images and randomly split the data set in two. For one of the subsets, we randomly chose a single volunteer annotation per image. For the other subset, we used the majority‐rule annotations. Finally, we fit a generalized linear model with *method* (single or triplicate scoring) and *trait* as predictors, *accuracy* as the binary response variable, and the logit link function. For these analyses, we also used the R statistical package (R Core Team, 2019) and the glm() function. Due to the complicated nature of accounting for the random effect of *volunteer* when dealing with majority rule annotations, we did not include this variable in these models.

In the previous analyses, we only used data from volunteers who scored 30 or more images. We also wanted to determine if those volunteers who produce few image annotations were better or worse than those who annotate many images. Three hundred forty‐two of the 392 (87%) volunteers who participated in the NfN expeditions for *Acer* and *Prunus* annotated fewer than 30 images. To examine this, we binned volunteers into five categories according to the total number of images each volunteer annotated: (1) one image annotated, (2) 2–5 images annotated, (3) 6–29 images annotated, (4) 30–100 images annotated, and (5) ≥101 images annotated. We then calculated an average accuracy score for the volunteers in each bin.

### Comparison of volunteer annotation methods

We added a column to the data sets indicating which study (in‐person volunteer or NfN) the annotation came from, and then the data were combined with their shared fields of genus, trait, and volunteer. Using accuracy as the binary response variable, we fit a GLMM for the data using the predictors *genus*, *trait*, and *study* as fixed effects, a random intercept effect for *volunteer*, and the logit link function. In particular, we were interested if *study* was a significant predictor, and whether in‐person volunteers were more accurate than NfN volunteers.

### Species analyses

In certain species of *Prunus* and *Acer*, some of the phenological traits, like flowers or fruits, are less visually obvious than for other species. Because of these differences, we hypothesized that species would affect annotation accuracy. To test this, we first removed species with fewer than 30 images in the two study data sets. We then fit a GLMM using *accuracy* as the binary response variable with the logit link function. We used the same fixed predictors described above for the in‐person and NfN studies, and for the random intercept effects, we included *volunteer* and added *species*. The final best‐fit models were determined by following the methodology described above for predictor and model selection.

## RESULTS

### Gold‐standard data set

A key result of our efforts is a gold‐standard, highly vetted data set of images of 3000 herbarium specimens of *Acer* and 2998 specimens of *Prunus*, each with annotations for unfolded leaves, fruits, and flowers. This data set was re‐evaluated after each independent attempt to score, whether from initial machine learning outputs or from the in‐person or NfN volunteers. This proved essential because each time we found a small, non‐zero percentage of records that were scored wrong in the “gold standard” and needed to be corrected. In addition, our volunteer annotation experiments revealed two images identified as *Prunus* that actually belonged to other genera. We removed both images from the final data set, resulting in 2998 records for *Prunus*. The final data set included 118 distinct scientific names for *Acer* and 212 for *Prunus*. The final version of this data set is available for download and re‐use at the Zenodo repository (Brenskelle et al., 2020).

### Are certain phenological traits or taxonomic groups easier to annotate than others?

**FIGURE 2:**
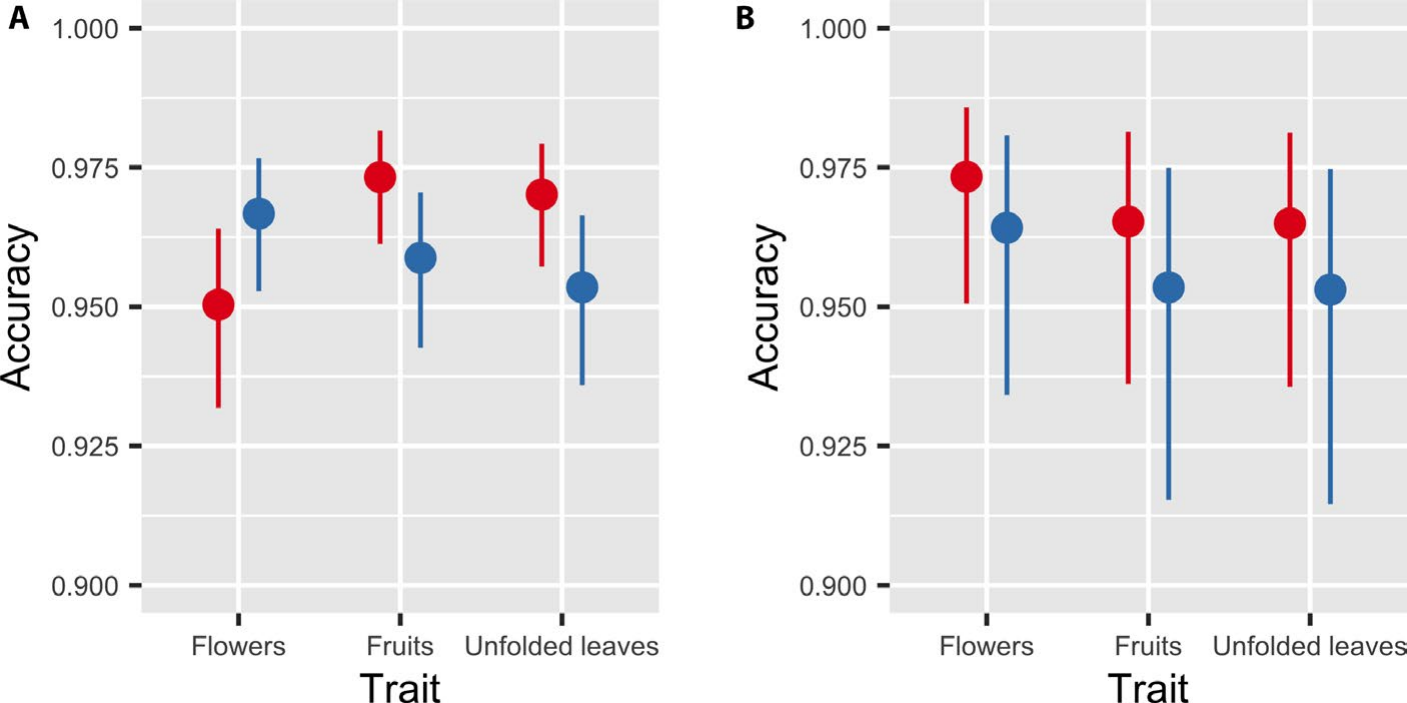
Predicted accuracy of in‐person volunteers (A) and Notes from Nature volunteers (B) given the plant genus and phenological trait. Error bars represent 95% confidence intervals.

In both the in‐person and NfN studies, we did find variation in volunteer annotation accuracy between the two genera and across the three phenological traits. In the in‐person volunteer study, the interaction effect between *genus* and *trait* was significant (*P* < 0.001), indicating that the difficulty of annotating phenological traits differed between the two genera. Flowers were more often correctly identified on specimens of *Prunus*, but for fruits and unfolded leaves, *Acer* was more often correct (Fig. 2A). For the online NfN study, the interaction effect between *genus* and *trait* was not significant. However, the main effects of *genus* and *trait* were significant (*P* < 0.001 for *genus* and *trait*). Volunteers were slightly more accurate at annotating *Acer* than *Prunus*, and for both genera, flowers were more accurately scored than either leaves or fruits (Fig. 2B).

The above models only considered genus‐level differences in annotation accuracy among volunteers. However, we noted in the development of the gold‐standard data set that some species of *Prunus* or *Acer* were more difficult than others. We tested this with models including *species* as a random effect. For these analyses, we excluded species with fewer than 30 images (see Methods). We found that *species* as a random effect explained a significant amount of the variation in annotation accuracy for both in‐person and NfN annotations (conditional *R* ^2^ [*R* ^2^c] = 0.104 for in‐person annotations, *R* ^2^c = 0.301 for NfN annotations; Table 2; Nakagawa and Schielzeth, 2013).

### How do differences among volunteers affect annotation accuracy?

**FIGURE 3:**
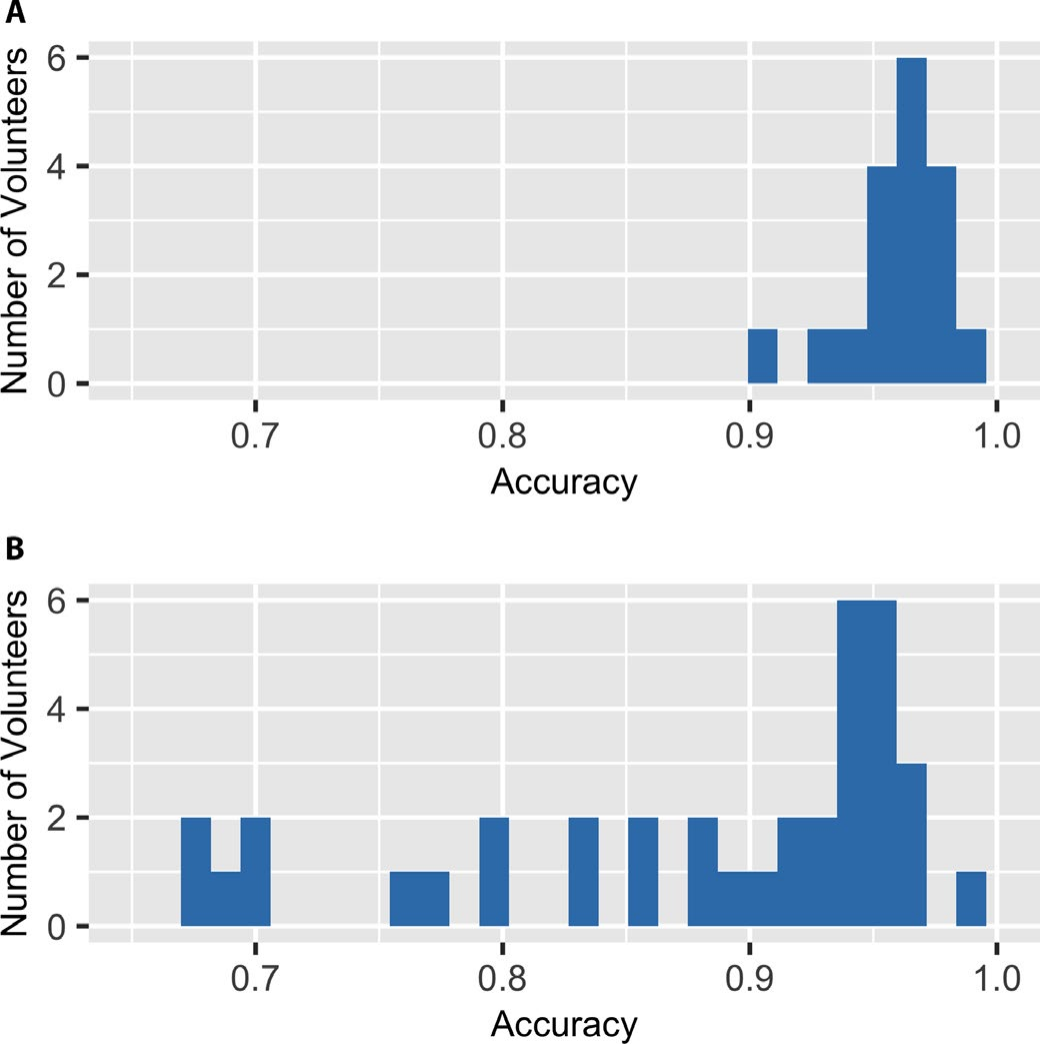
Histograms of volunteer accuracy for the in‐person (A) and Notes from Nature (B) studies. Notes from Nature volunteers who annotated fewer than 30 images were excluded.

We found that volunteers strongly vary in their capacity to perform annotation tasks, with a greater range of variation among NfN volunteers than in‐person volunteers (Fig. 3). Our best models for both the in‐person and NfN experiments included *volunteer* as a random effect, and in both cases, *volunteer* explained much of the variation in annotation accuracy (*R* ^2^c = 0.062 for in‐person annotations, *R* ^2^c = 0.274 for NfN annotations; Table 1). We found that neither previous botanical expertise (*P* = 0.063) nor career level (*P* = 0.105) had a significant effect on the annotation accuracy of our in‐person volunteers, although previous botanical expertise was nearly significant. Simply put, volunteers differ in capability in annotation tasks irrespective of any a priori grouping we assigned.

### Does speed of annotation matter for quality; e.g., are slower annotators more accurate?

During the in‐person volunteer study, we noticed that there was considerable disparity in how much time each of the volunteers spent on the annotation task, even though each volunteer was given the same number of images to score. Some volunteers took as little as 25 minutes to score all 600 images, while at least one volunteer spent over 160 minutes. Much to our surprise, we did not find that time spent had a significant effect on annotation accuracy (*P* = 0.602), at least in the case of the in‐person study. We found similar results for the NfN study, where the time spent per image was also non‐significant (*P* = 0.261). Overall, our findings do not indicate that time spent annotating has a significant negative or positive impact on accuracy.

### Do annotators improve with practice or fatigue with more effort?

We were interested to see how much annotation accuracy improved, if at all, with increased effort or practice, defined by the total number of images annotated. For the NfN study, we found that annotation accuracy improved as the total number of images a volunteer annotated increased (*P* = 0.01, coefficient of *image_count* = 0.598; 95% confidence interval = 0.061–1.132).

Additionally, we were interested in how the volunteers who scored fewer than 30 images performed. Were the volunteers who annotated only a single image just as accurate as volunteers who did more? Table 3 shows the results of our efforts (methods described above) to explore this. In general, we found that the accuracy of online volunteers improved as the number of images scored increased. This was not tested in the in‐person study because all volunteers annotated the same number of images.

**Table 3:** The distribution of the number of images scored by all 392 Notes from Nature volunteers, and the percent of images each group scored correctly.

No. of images scored	No. of volunteers	Percent correct
1	70	82%
2–5	144	82%
6–29	128	86%
30–100	27	88%
101+	23	96%

### Is there a difference in annotation accuracy for Notes from Nature versus in‐person volunteers?

**FIGURE 4:**
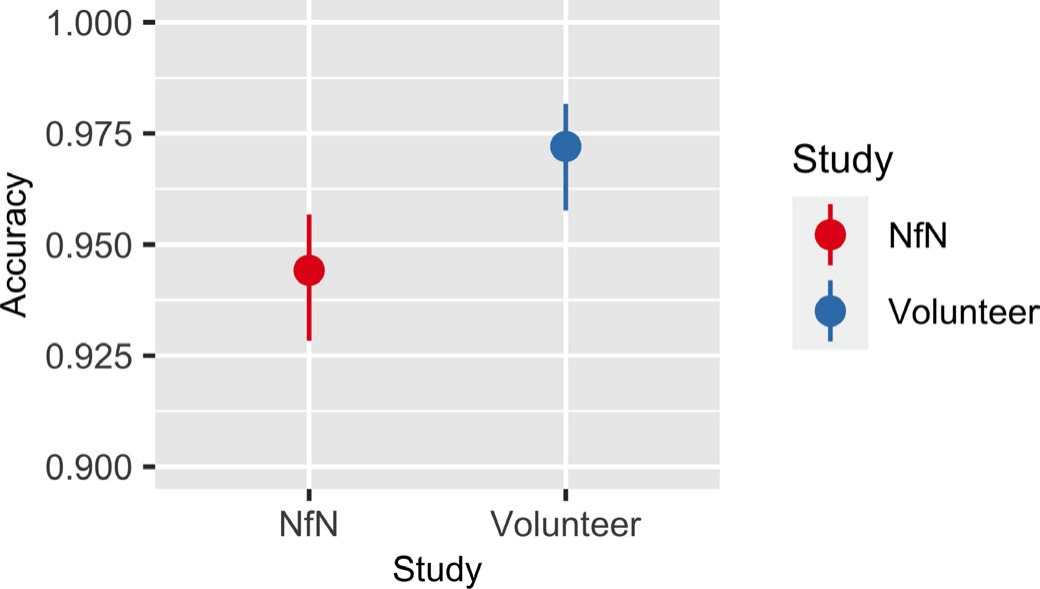
Predicted accuracy for the in‐person and Notes from Nature (NfN) studies. Error bars represent 95% confidence intervals.

We found a significant difference in accuracy between the two annotation methods (*P* = 0.004). Although both in‐person and NfN volunteers on average had over 90% accuracy, in‐person volunteers were, on average, 2.8% more accurate than their online NfN counterparts (Fig. 4).

### Does majority‐rule scoring increase accuracy?

**FIGURE 5:**
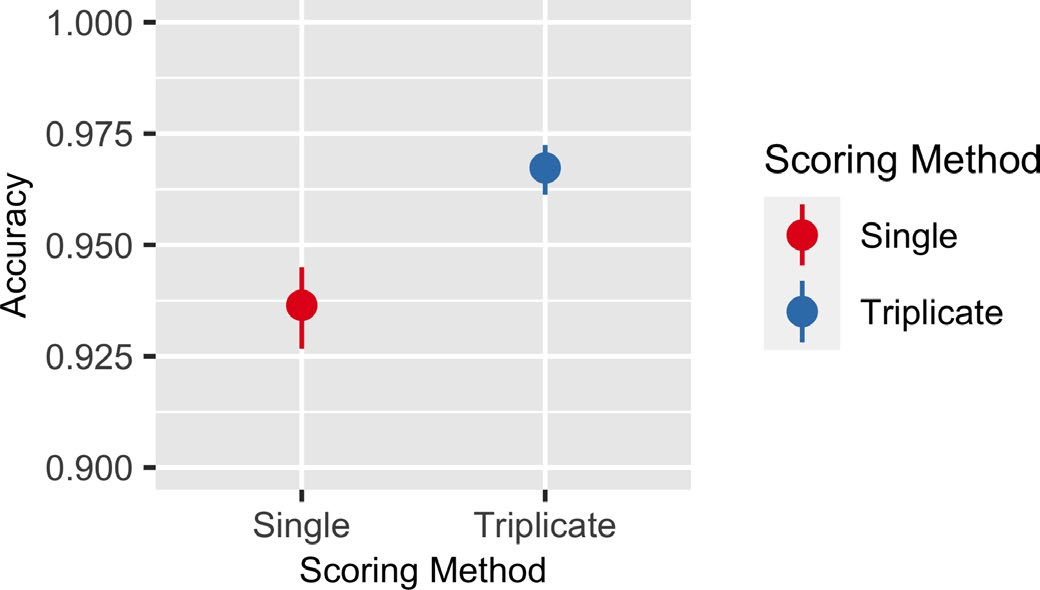
Predicted accuracy for majority‐rule scorings (triplicate scoring; two out of three annotations) versus single classifications from the Notes from Nature study. Error bars represent 95% confidence intervals.

We also wanted to determine if this majority‐rule approach improves accuracy compared to simply relying on one classification. We found that, when compared to single NfN classifications, the majority rule of triplicate scoring in NfN increased the annotation accuracy from 93.5% to 96.5% (*P* < 0.001; Fig. 5).

## DISCUSSION

### Comparison of the two methods

Overall, both methods performed well (accuracies generally >94%), but we found that in‐person volunteers gave more accurate annotations. We also found a large variation in volunteer accuracy across the two studies. Although our modeling efforts were unable to pinpoint exactly what makes someone a good image annotator, there was greater variation in accuracy among the NfN volunteers (Fig. 4). The key factors that we think may account for this disparity are a lack of in‐person training for NfN volunteers, differences in the usage of the genus‐specific handbooks (which could also be related to differences in training), differences in image size/quality, and the control or lack thereof for the number of images scored by each volunteer.

We provided training for our in‐person volunteers that included explanation of the annotation tasks, a discussion of the phenological traits and how to interpret the terminology, and discussion of examples included in the genus‐specific annotation handbooks. This training took approximately five minutes, and then we answered questions the volunteers had before they began annotation. In‐person volunteers were provided with both printed and digital copies of the handbooks and we asked each volunteer to take some time to read through the handbooks before annotating any images. In contrast, although NfN volunteers were provided with digital versions of the handbooks, we could not provide NfN volunteers with any direct training and we had no control over whether they even looked at the handbooks, let alone used them while annotating images.

Although we did not make formal observations noting the use of the annotation handbooks by the in‐person volunteers, the handbooks include examples of difficult specimens (either due to the species’ morphology or the phenological trait being scored, because plant phenologies can be hard to discretize into stages even if commonly scored that way; e.g., Lorieul et al., 2019) and detailed reference material, and we noticed that at least some in‐person volunteers referred to them often, especially at the beginning of annotation sessions. Our statistical analyses indicated that the effect of *species* was not as strong in the in‐person study as in the NfN study (Table 2). This may be because in‐person volunteers had the handbook physically in front of them and were more likely to use it to help with difficult specimens, while NfN volunteers could skip even looking at the handbooks. We did use the NfN Talk feature to engage with online volunteers in order to answer general questions during the annotation process. These differences in training and use of the handbook information may partially explain both the greater variance in NfN accuracy when compared to the in‐person volunteers and greater species variation.

In addition, as mentioned above, the NfN platform limits image size to 1 MB. Therefore, we had to scale down some of the herbarium images to meet these requirements, and the NfN volunteers were not always able to view full‐scale images like the in‐person volunteers. However, we did become aware via the NfN Talk feature that some of the NfN volunteers were locating full‐size specimen images from other online sources. These differences between access to full‐size images between the in‐person and NfN volunteers, and the differences between the NfN users who sought out full‐size images and those who did not, may further explain the variation in volunteer accuracy.

As shown in Table 3, volunteer accuracy increased with the number of images scored. In the in‐person study, we were able to keep the number of images annotated by each volunteer equal, possibly leading to greater consistency in volunteer accuracy (Fig. 3). This not only allows volunteers to gain annotation experience, but also prevents an inaccurate volunteer from annotating a disproportionately large number of images in the data set. In the NfN study, 342 volunteers scored fewer than 30 images, whereas five volunteers scored 600 or more images, the same number scored by all 18 in‐person volunteers.

We were surprised to find that neither prior botanical experience, academic career level, nor time spent per image were predictive of accuracy for in‐person volunteers. The relative unimportance of prior botanical experience and academic career level could be evidence that good volunteer training can overcome initial differences in these two factors. The result for time spent per image is harder to explain. In our own experience, we found that careful examination of images sometimes revealed subtle phenological information on the herbarium sheets. However, such images are relatively rare, which could mean that rapid classification can still yield relatively high accuracy.

### Best‐practice recommendations

Using information from our studies, here we provide a set of best‐practice recommendations for image annotation projects. Although our project used herbarium specimen images to generate phenological image annotations, these same guidelines could be used for any type of image annotation project on any group of interest.

1. Identify particularly difficult species/classes of traits to annotate before launching the annotation project. This is important for developing unambiguous, broadly applicable annotation criteria, and for choosing examples to include in training materials.
2. Develop informative training materials. Although there may be multiple unmeasured factors determining baseline volunteer annotation accuracy, our findings indicate that non‐expert volunteers are trainable. This places a premium on carefully constructed, easy‐to‐use training materials that explain the annotation task and provide informative, well‐documented examples.
3. If image size has to be reduced, balance downsampling and image compression to maximize the visual quality of the images. For example, one could use the algorithm presented in the Methods to reduce image size while preserving as much image quality as possible.
4. Perform in‐person annotations, in absence of other time and resource constraints. We found that in‐person annotations were significantly more accurate than annotations from an online community science platform. However, we note that the differential in quality is not that high, so public participation using online platforms is a perfectly reasonable alternative.
5. Perform majority‐rule scoring and/or distribute effort evenly among volunteers. To mitigate volunteer variance, especially if using a community science platform, we recommend majority‐rule scoring, ensuring an equal distribution of images per volunteer, and/or setting a minimal number of classifications that a volunteer must complete to be included in the final data set. These efforts guard against a single, chronically inaccurate volunteer from swamping the data set, and allow volunteers to gain experience with the annotation task, thereby increasing their accuracy. Table 3 can be used as guidance for selecting a threshold, but we suggest removing data from volunteers who score five images or fewer.
6. To implement majority‐rule scoring, we recommend beginning by obtaining two annotations for each image. Images for which these first two annotations agree do not require any further annotation effort. Images for which the first two annotations disagree should be annotated independently a third time to generate a majority‐rule annotation.

Considering the best practices outlined above and the results of our annotation experiments, we conclude that an optimal (in terms of annotation accuracy) image annotation project will use in‐person volunteers, distribute annotation effort evenly among all volunteers, and calculate majority‐rule annotations for each image based on triplicate scoring. Nevertheless, our results also suggest it is quite possible to generate high‐quality data even when some best‐practice recommendations are not implemented. Across all species and traits, we recovered relatively high‐quality data using both annotation methods, with overall accuracies above 94%. This suggests that with careful project design, including well‐defined trait definitions and informative visual training materials, volunteer image annotations can lead to usable data sets for both scientific research and for training machine learning algorithms. Implementation of these best‐practice recommendations will help the scientific community leverage rapidly expanding specimen image resources for a range of novel purposes.

## AUTHOR CONTRIBUTIONS

L.B., B.J.S., and R.P.G. conceived of this work together. B.J.S. performed the image and image metadata collection, data cleaning, and image modification for Notes from Nature. Initial annotations and reconciliation was done by L.B., B.J.S., and R.P.G. B.J.S. developed the image annotation tool. M.D. formatted and created the Notes from Nature expeditions. Data analysis was done by L.B. L.B. led writing of the manuscript with help from B.J.S. and R.P.G., and all authors helped with editing.

## Supporting information


**APPENDIX S1.** Herbarium scoring volunteer handbook for *Acer*.

**APPENDIX S2.** Herbarium scoring volunteer handbook for *Prunus*.

**APPENDIX S3.** Herbarium specimen machine learning survey.

## Data Availability

The data sets used for this study are archived on the Zenodo repository (Brenskelle et al., 2020).
