# Corrigendum

**DOI:** 10.1002/aps3.11381

**Published:** 2020-07-31

In our paper, “Maximizing human effort for analyzing scientific images: A case study using digitized herbarium sheets,” Figure 2 was published without the legend. We apologize for the error. The published article (full‐text and PDF) has been corrected.

**Figure 2:**
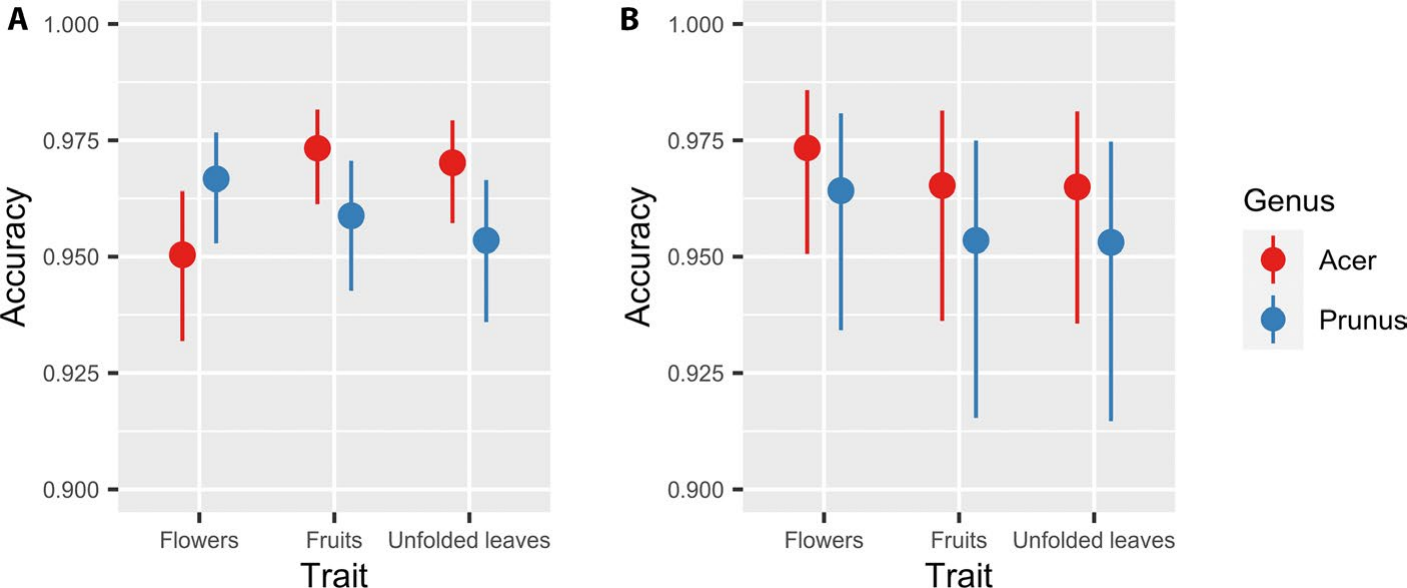
Predicted accuracy of in‐person volunteers (A) and Notes from Nature volunteers (B) given the plant genus and phenological trait. Error bars represent 95% confidence intervals.
