# Supplementary material for: Maximizing human effort for analyzing scientific images: A case study using digitized herbarium sheets
Source: Appl Plant Sci. 2020 Jul 1;8(6):e11370. doi: 10.1002/aps3.11370 (PMC7328657; doi:10.1002/aps3.11370)
Supplement: Supplementary file 1 — APPENDIX S1. Herbarium scoring volunteer handbook for Acer. [file APS3-8-e11370-s001.pdf]

doi:10.1002/aps3.11370

Herbarium Scoring Volunteer Handbook

Acer

Flowers, Fruits and Unfolded Leaves

Collated by Laura Brenskelle

September 2018

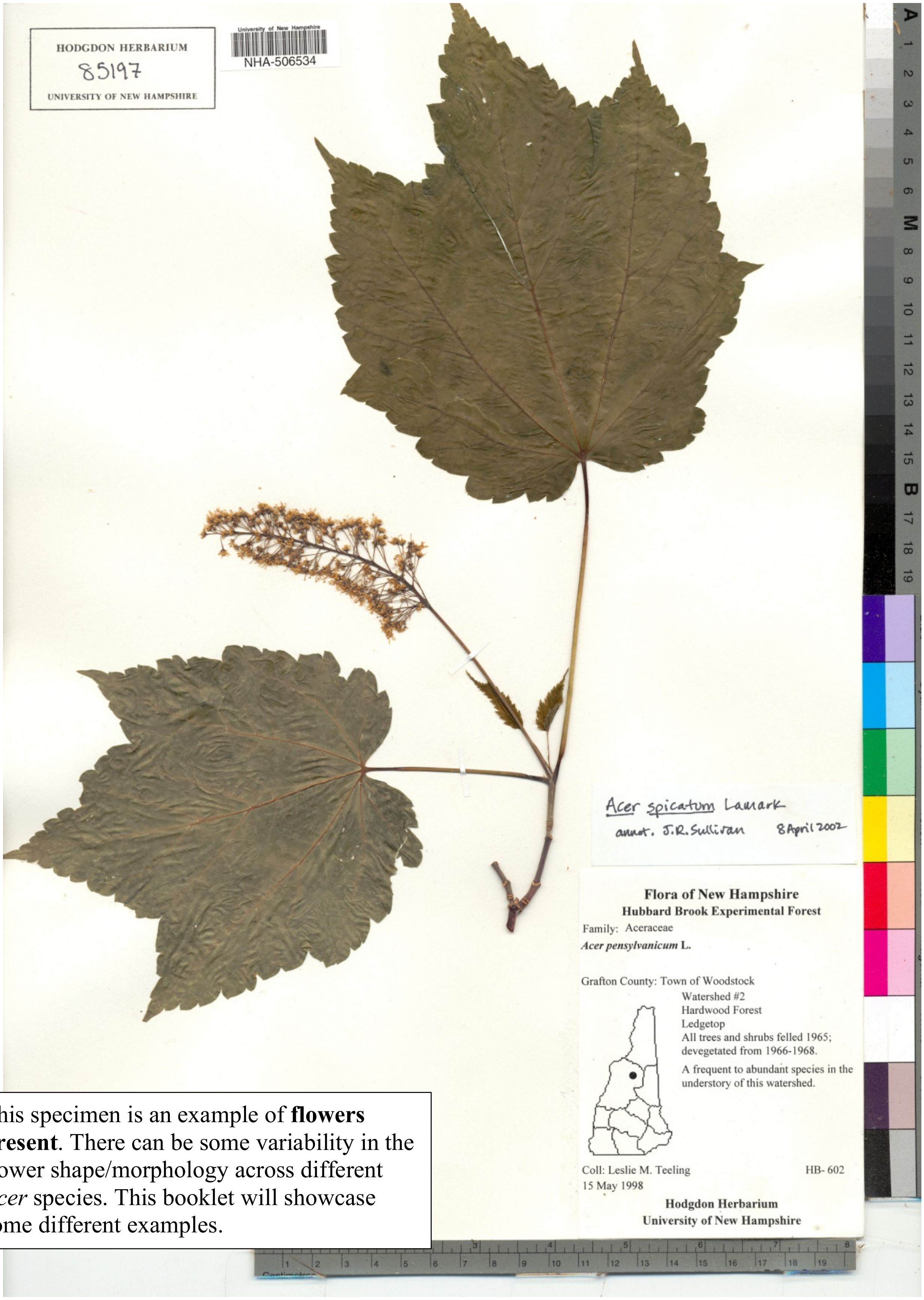

This specimen is an example of **flowers present**. There can be some variability in the flower shape/morphology across different *Acer* species. This booklet will showcase some different examples.

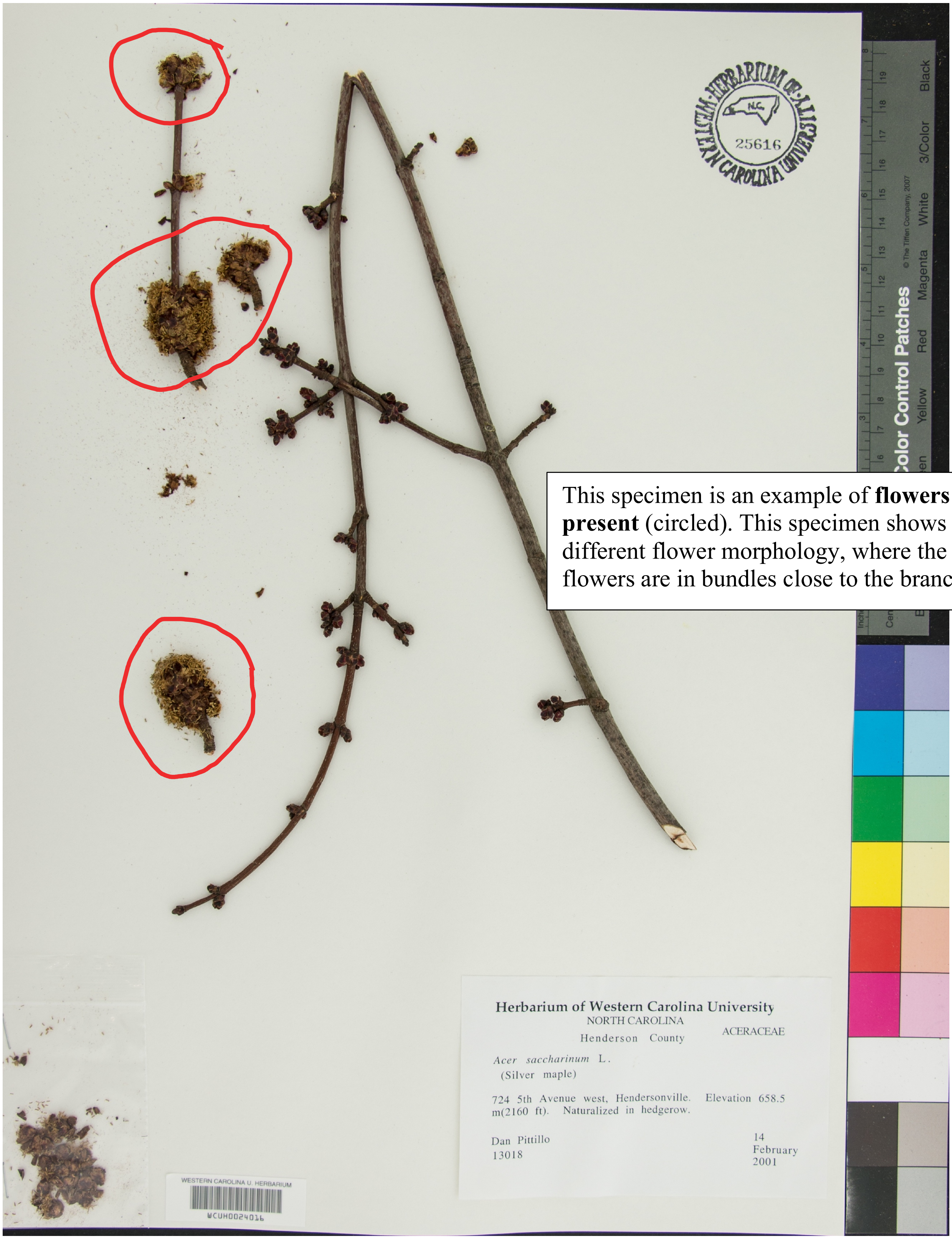

This specimen is an example of **flowers present** (circled). This specimen shows a different flower morphology, where the flowers are in bundles close to the branch.

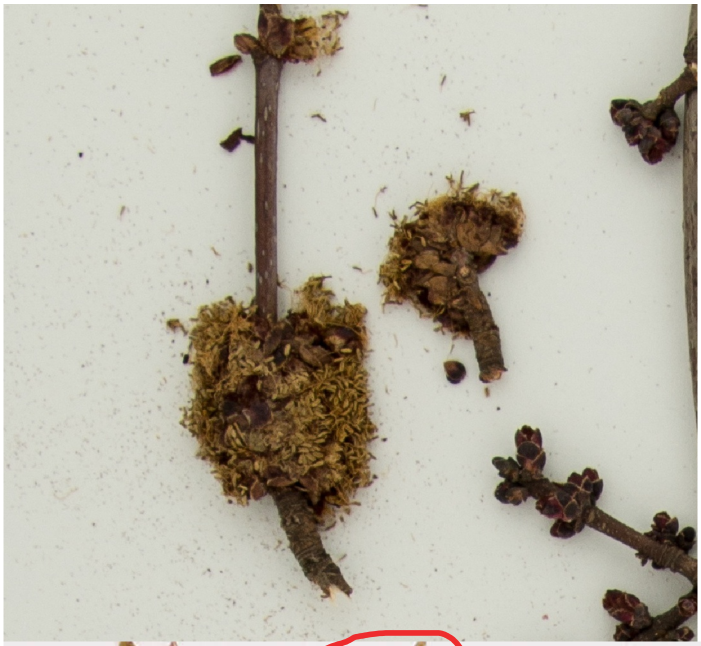

This image (left) is a zoomed in view of some of the flower clusters shown on the previous page. This is an example of **flowers present** on a specimen.

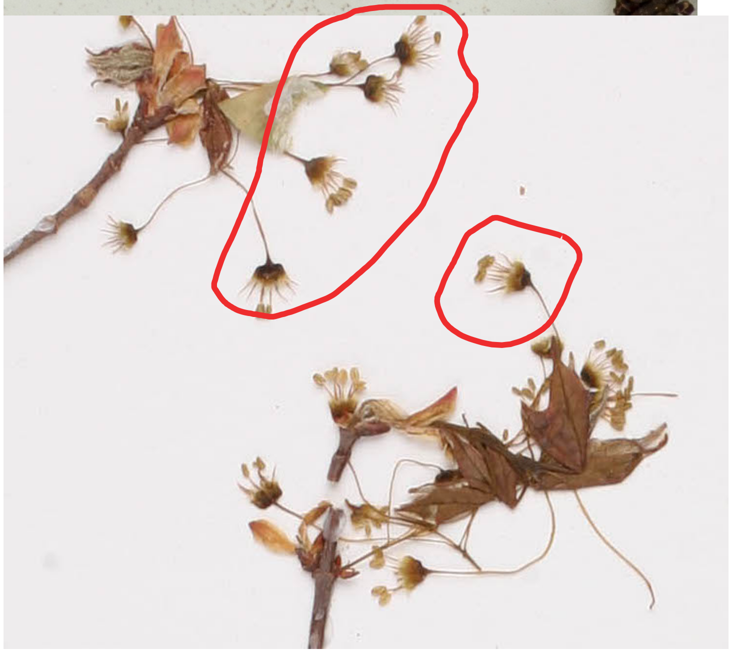

This image (left) is an example of **flowers present**. The flowers (circled) have a slightly different morphology than the last two examples shown.

DOI

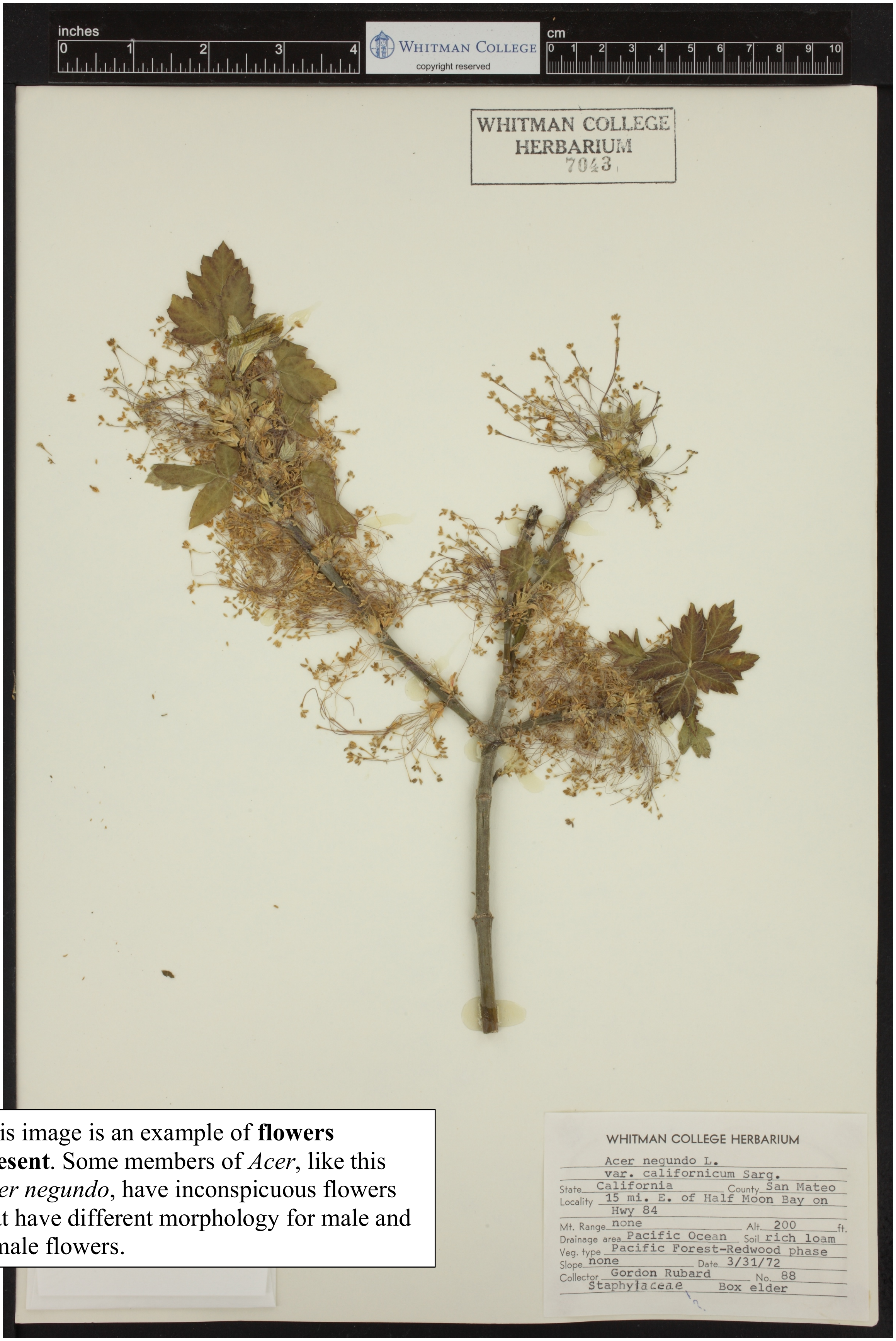

This image is an example of **flowers present**. Some members of *Acer*, like this *Acer negundo*, have inconspicuous flowers that have different morphology for male and female flowers.

DOI

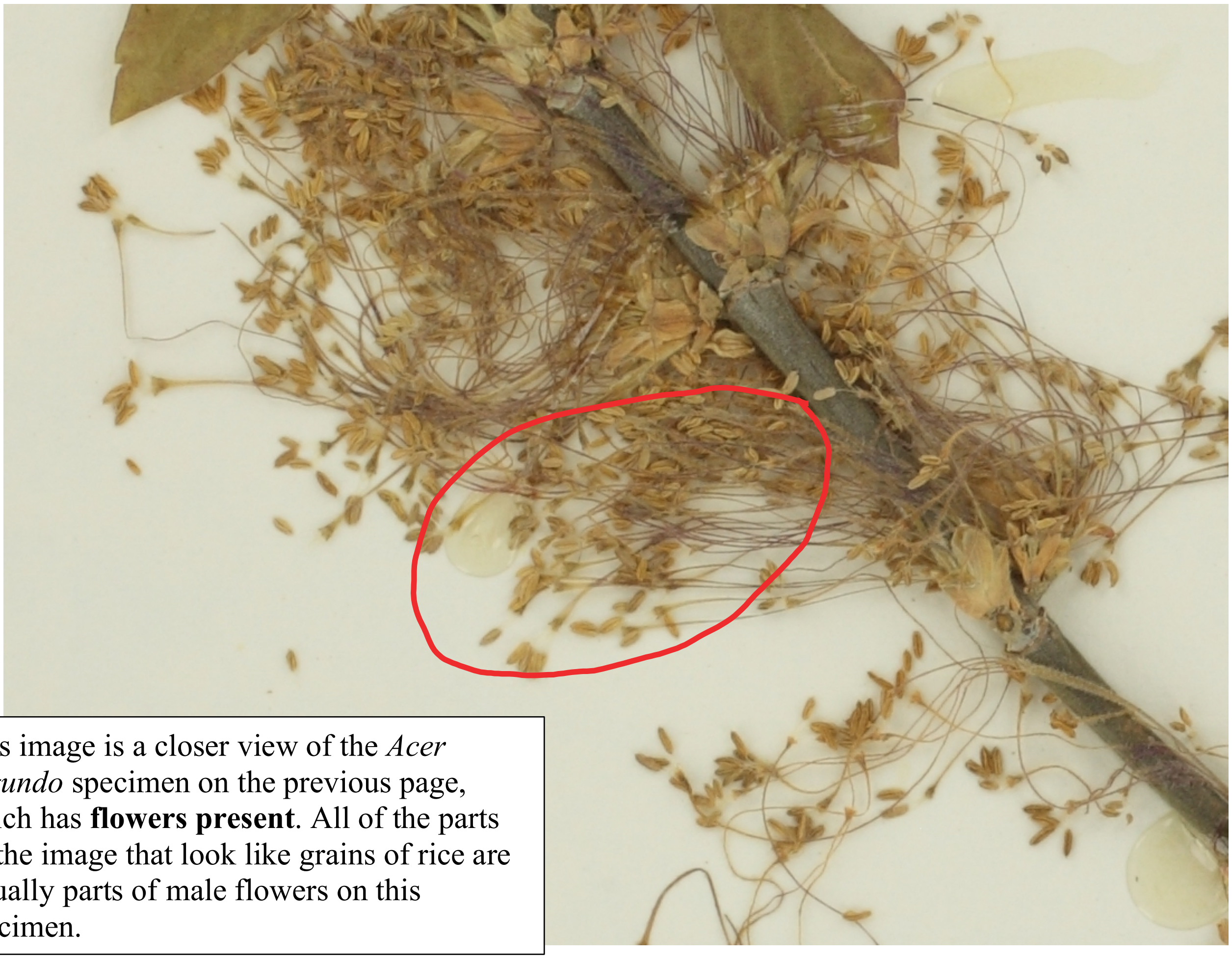

This image is a closer view of the *Acer negundo* specimen on the previous page, which has **flowers present**. All of the parts on the image that look like grains of rice are actually parts of male flowers on this specimen.

On the following pages, we will give you some anatomical drawings that we found helpful, given the variability in flowers across the genus. Note that these drawings do not include every species of *Acer*.

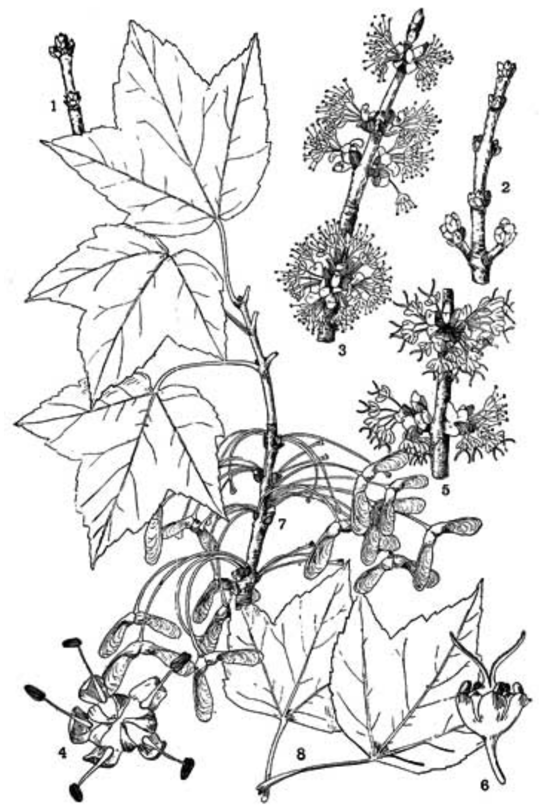

Acer rubrum[1]

1. Leaf-buds.
2. Flower-buds.
3. Branch with male flowers.
4. Male flower.
5. Branch with male and female flowers.
6. Female flower.
7. Fruiting branch.
8. Variant leaves.

DOI

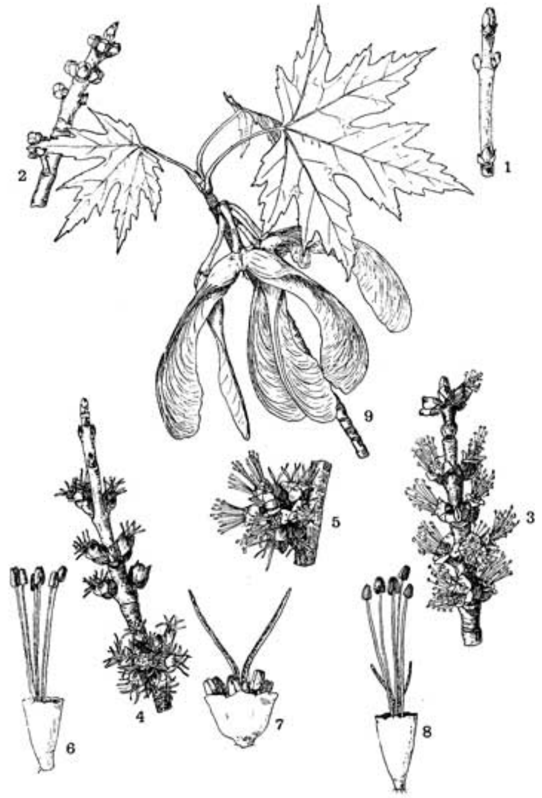

Acer saccharinum[1]

1. Leaf-buds.
2. Flower-buds.
3. Branch with male flowers.
4. Branch with female flowers.
5. Branch with male and female flowers.
6. Male flower.
7. Female flower.
8. Perfect flower.
9. Fruiting branch.

DOI

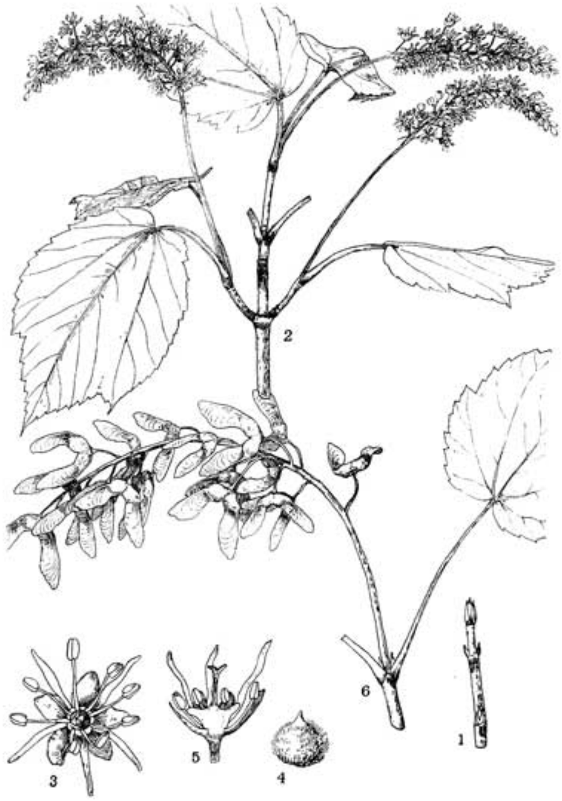

Acer spicatum[1]

1. Winter buds.
2. Flowering branch.
3. Male flower.
4. Abortive ovary in male flower.
5. Female flower with part of the perianth and stamens removed.
6. Fruiting branch.

DOI

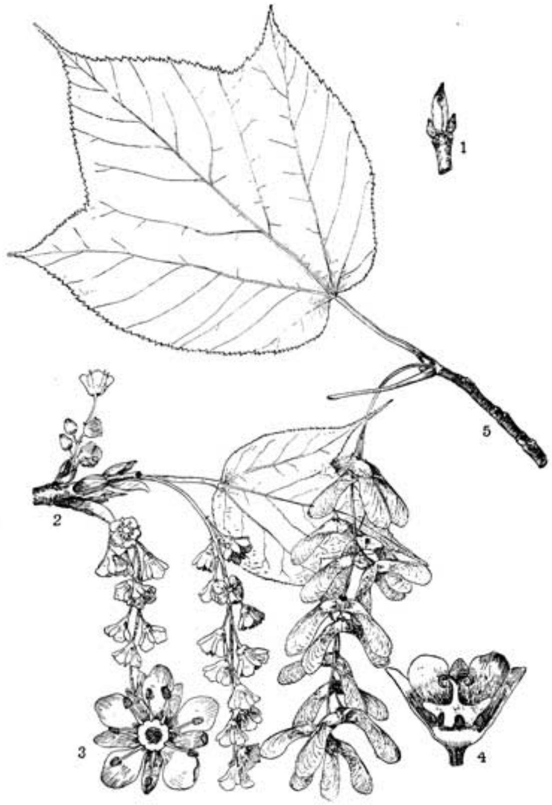

Acer pensylvanicum[1]

1. Winter buds.
2. Flowering branch.
3. Male flower.
4. Female flower with part of the perianth removed.
5. Fruiting branch.

DOI

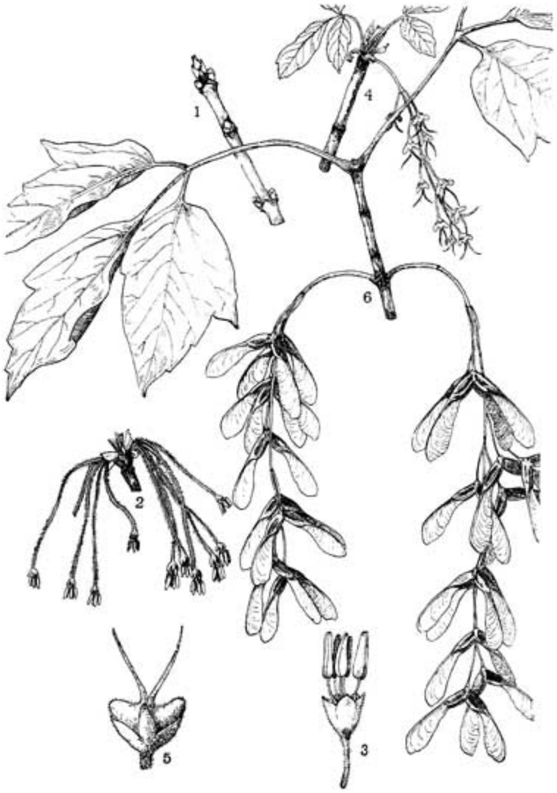

Acer negundo[1]

1. Winter buds.
2. Branch with male flowers.
3. Male flower.
4. Branch with female flowers.
5. Female flower.
6. Fruiting branch.

DOI

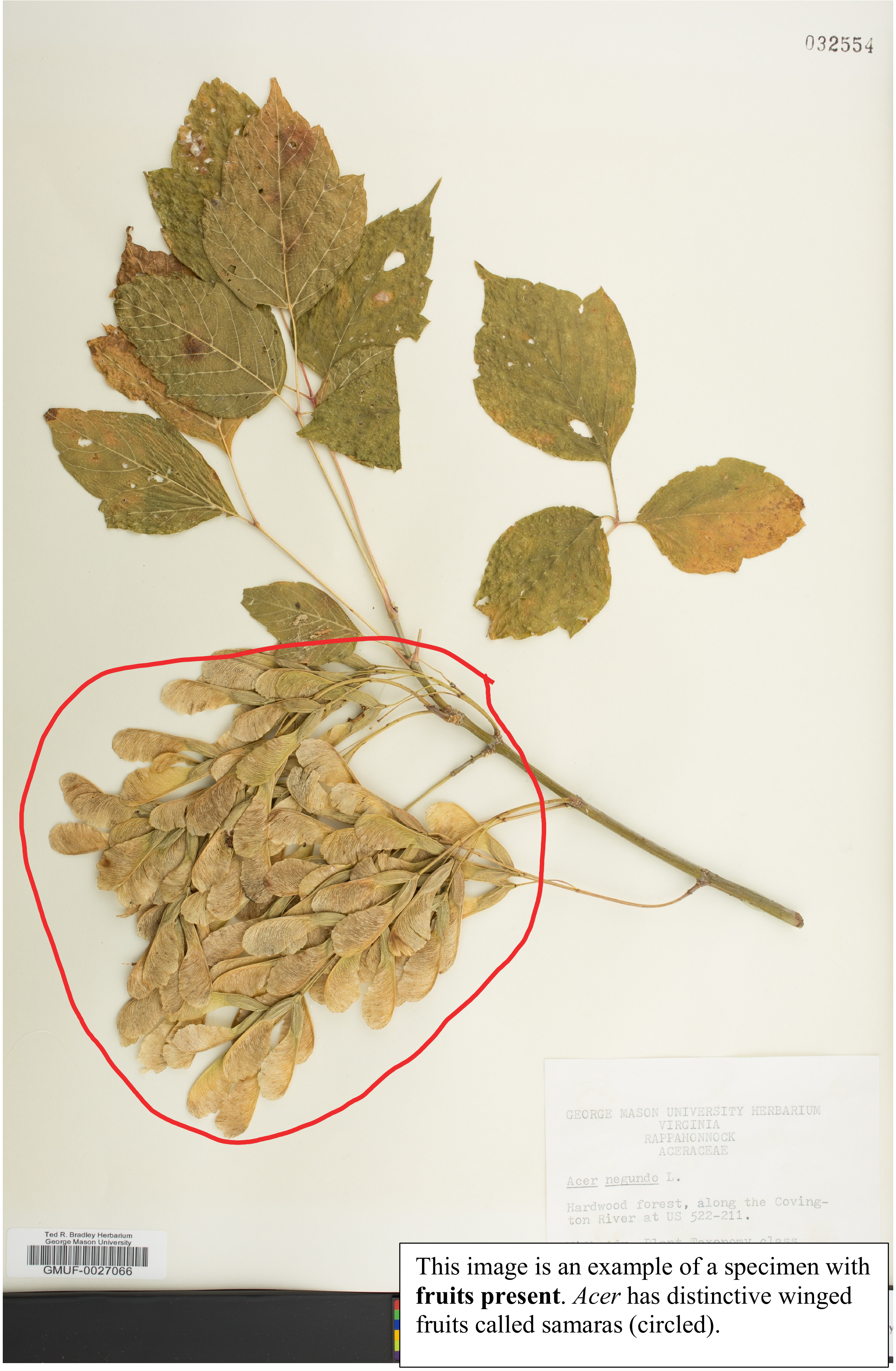

This image is an example of a specimen with **fruits present**. *Acer* has distinctive winged fruits called samaras (circled).

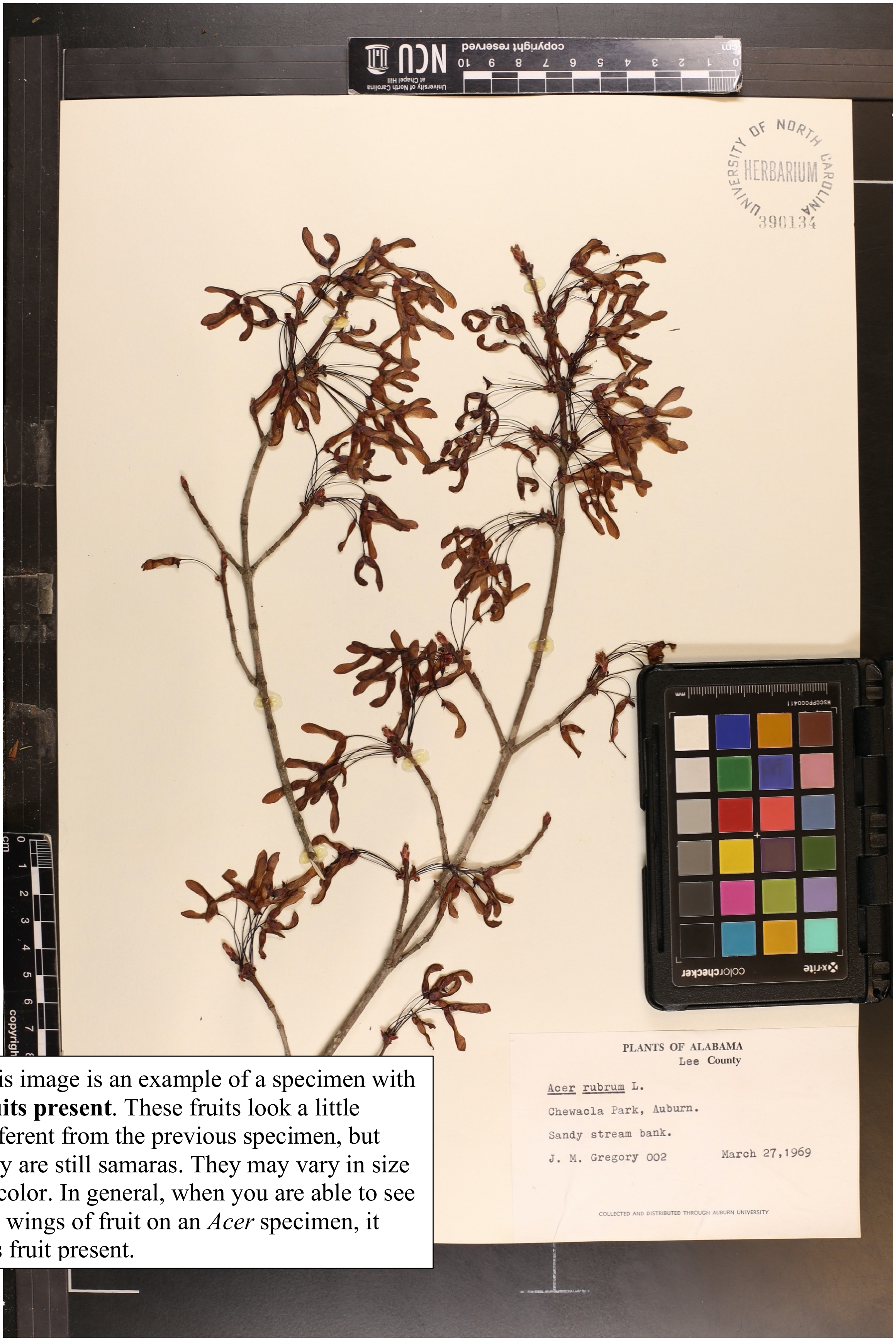

This image is an example of a specimen with **fruits present**. These fruits look a little different from the previous specimen, but they are still samaras. They may vary in size or color. In general, when you are able to see the wings of fruit on an *Acer* specimen, it has fruit present.

DOI

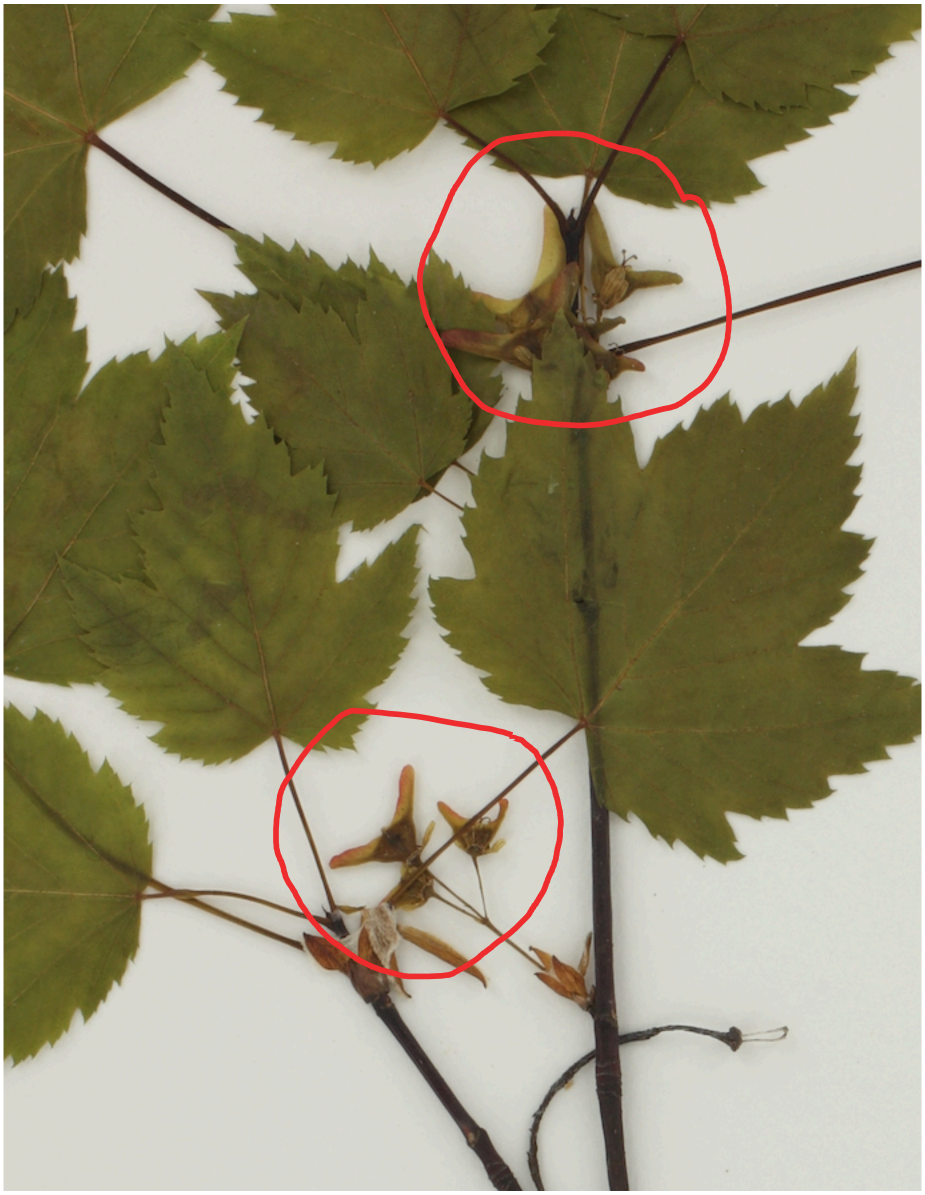

This image is an example of a specimen with **fruits present**. These are immature fruits, and for some of them you may even still be able to see floral structures present, but the wings are beginning to develop, and thus, they are scored as fruit present.

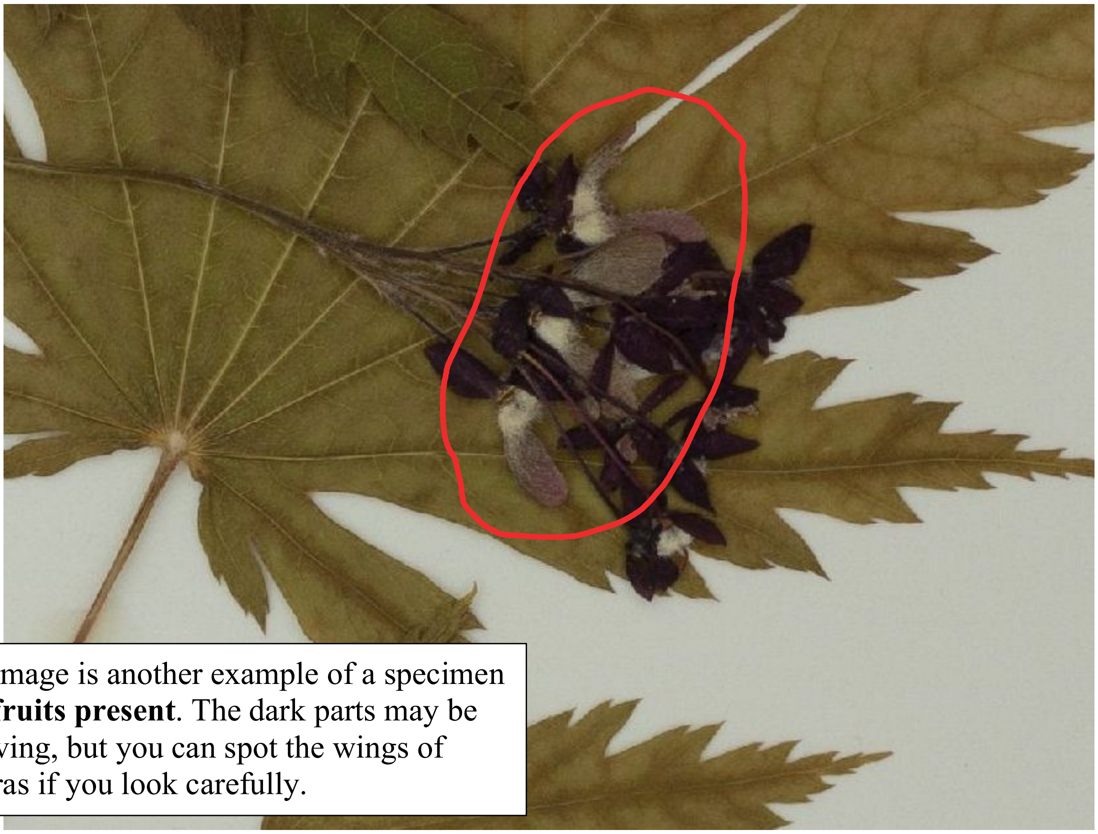

This image is another example of a specimen with **fruits present**. The dark parts may be deceiving, but you can spot the wings of samaras if you look carefully.

DOI

This image is another example of a specimen with **fruits present**. There are flowers present at the tip of the inflorescence, but the wings of immature fruit are present sticking out of some flowers at the base.

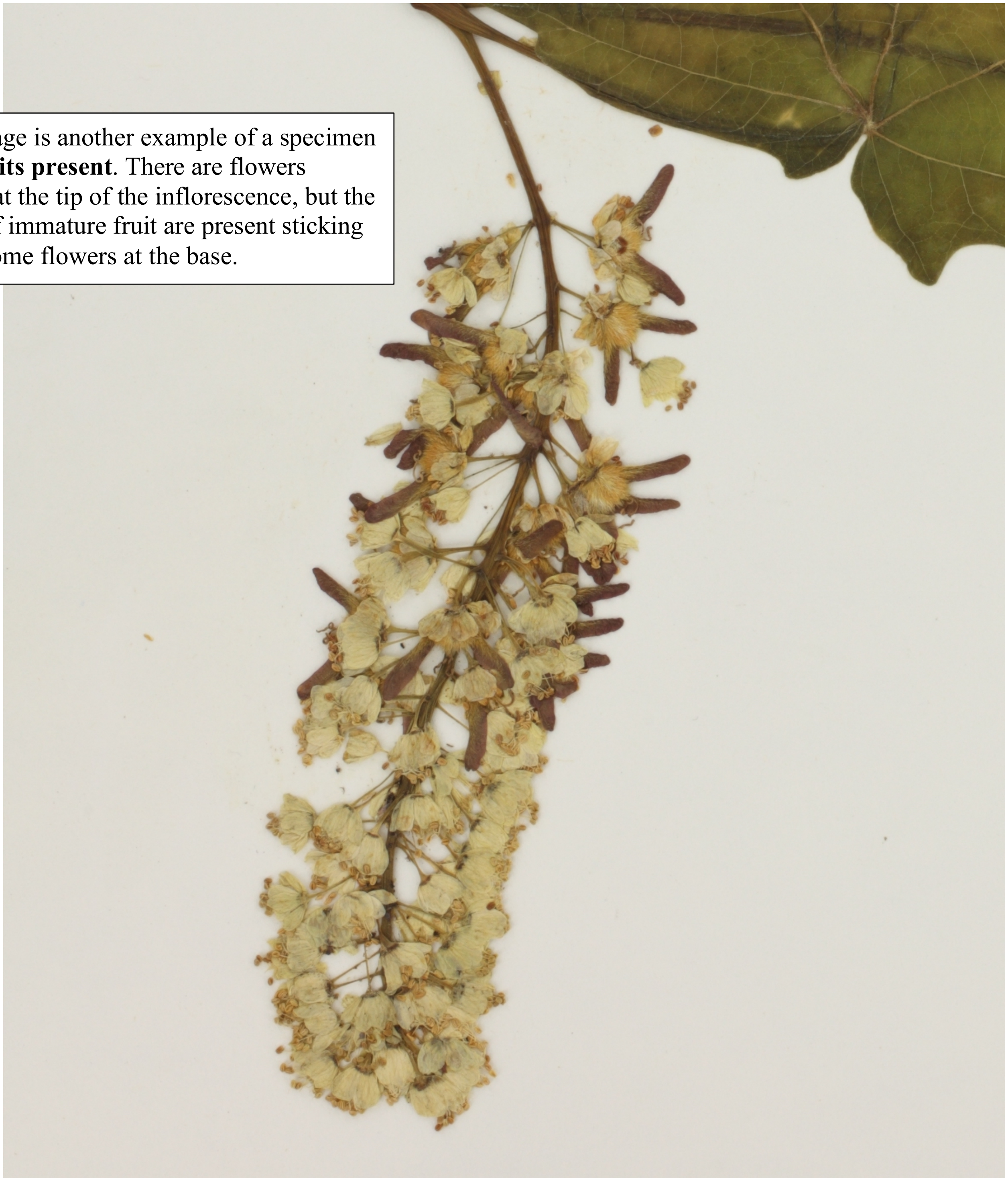

DOI

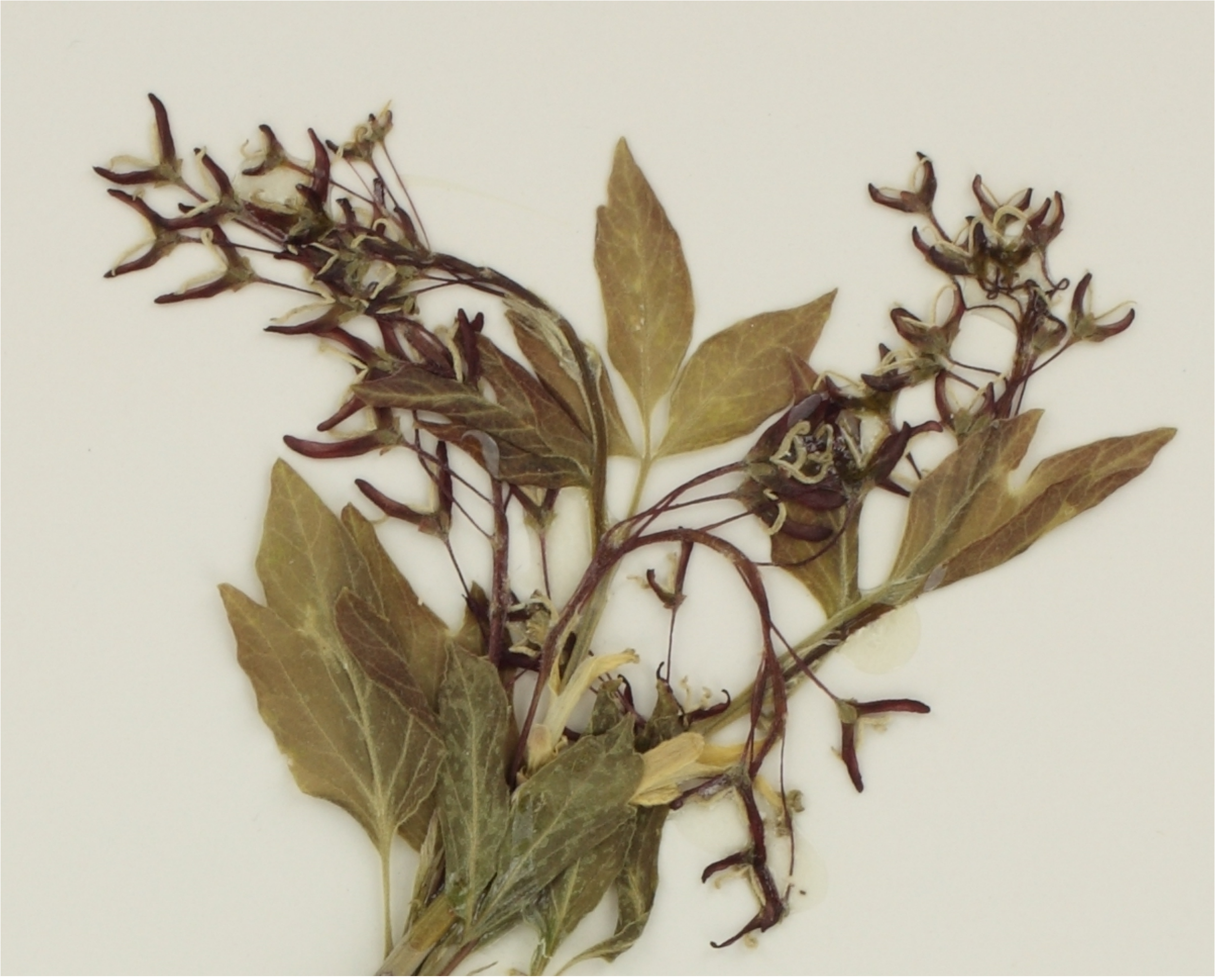

This image is an example of a specimen with **fruits present**. These are the female flowers of *Acer negundo*, and although floral parts may be present, the maturing wings of the samaras are visibly sticking out of the flowers.

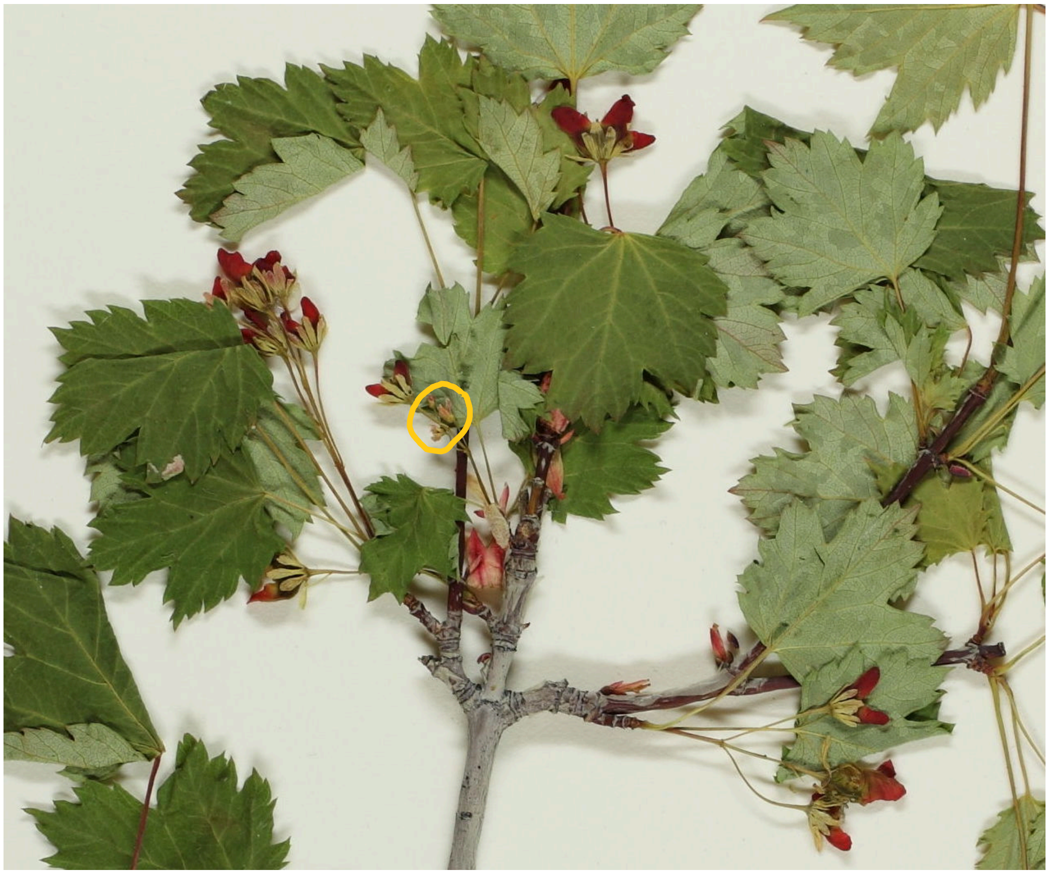

This image is an example of a specimen with **fruits present**. This is *Acer glabrum*, and the red parts are actually wings of samaras. The are some flowers present (circled in yellow), but they are not the red parts present on the specimen.

DOI

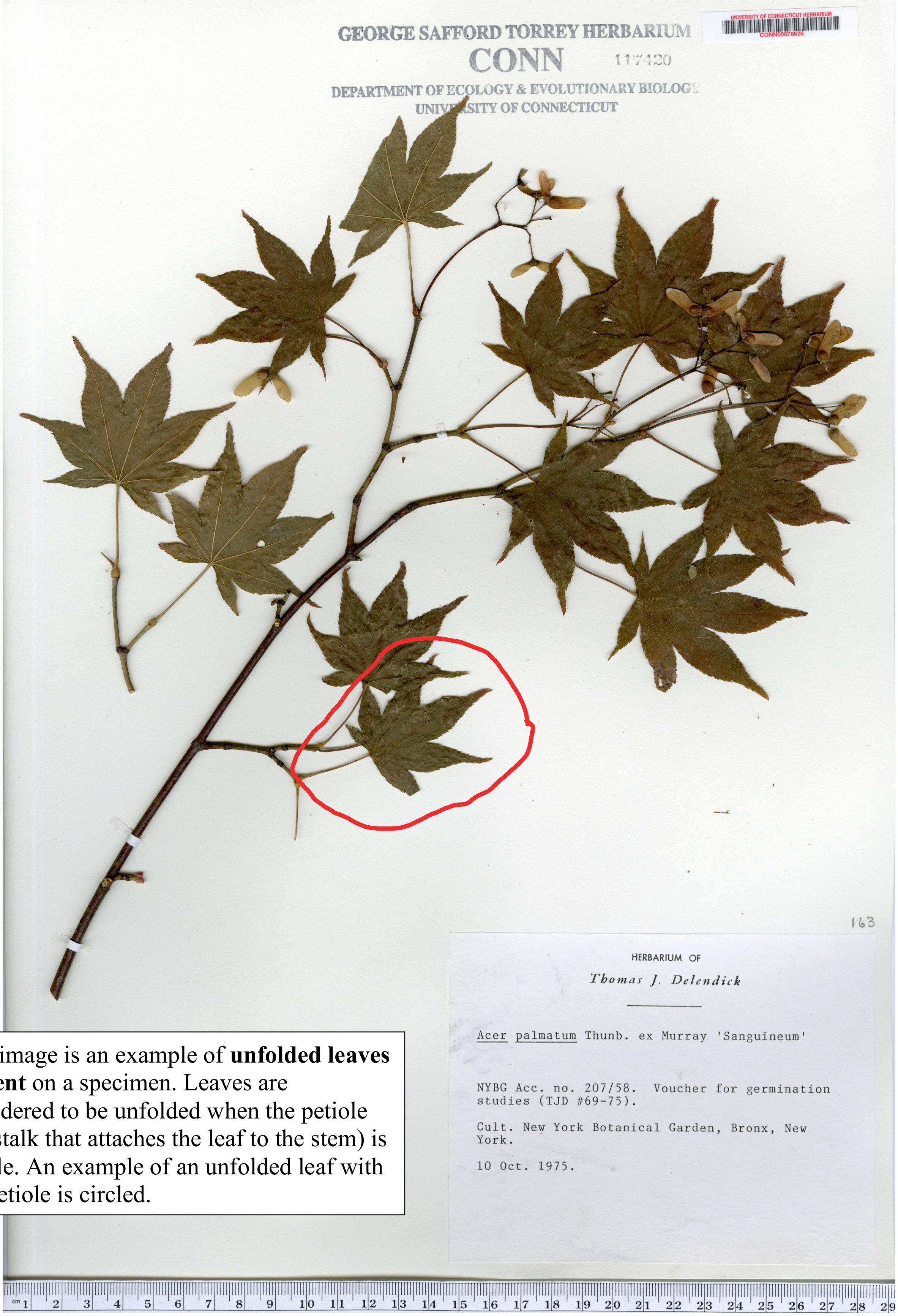

This image is an example of **unfolded leaves present** on a specimen. Leaves are considered to be unfolded when the petiole (the stalk that attaches the leaf to the stem) is visible. An example of an unfolded leaf with the petiole is circled.

References

[1]Brooks HM, Dame LL. Handbook of the Trees of New England, with Ranges Throughout the United States and Canada. Boston: Ginn; 1901.
