# Supplementary material for: Maximizing human effort for analyzing scientific images: A case study using digitized herbarium sheets
Source: Appl Plant Sci. 2020 Jul 1;8(6):e11370. doi: 10.1002/aps3.11370 (PMC7328657; doi:10.1002/aps3.11370)

doi:10.1002/aps3.11370

Herbarium Scoring Volunteer Handbook

Prunus

Flowers, Fruits and Unfolded Leaves

Collated by Laura Brenskelle

September 2018

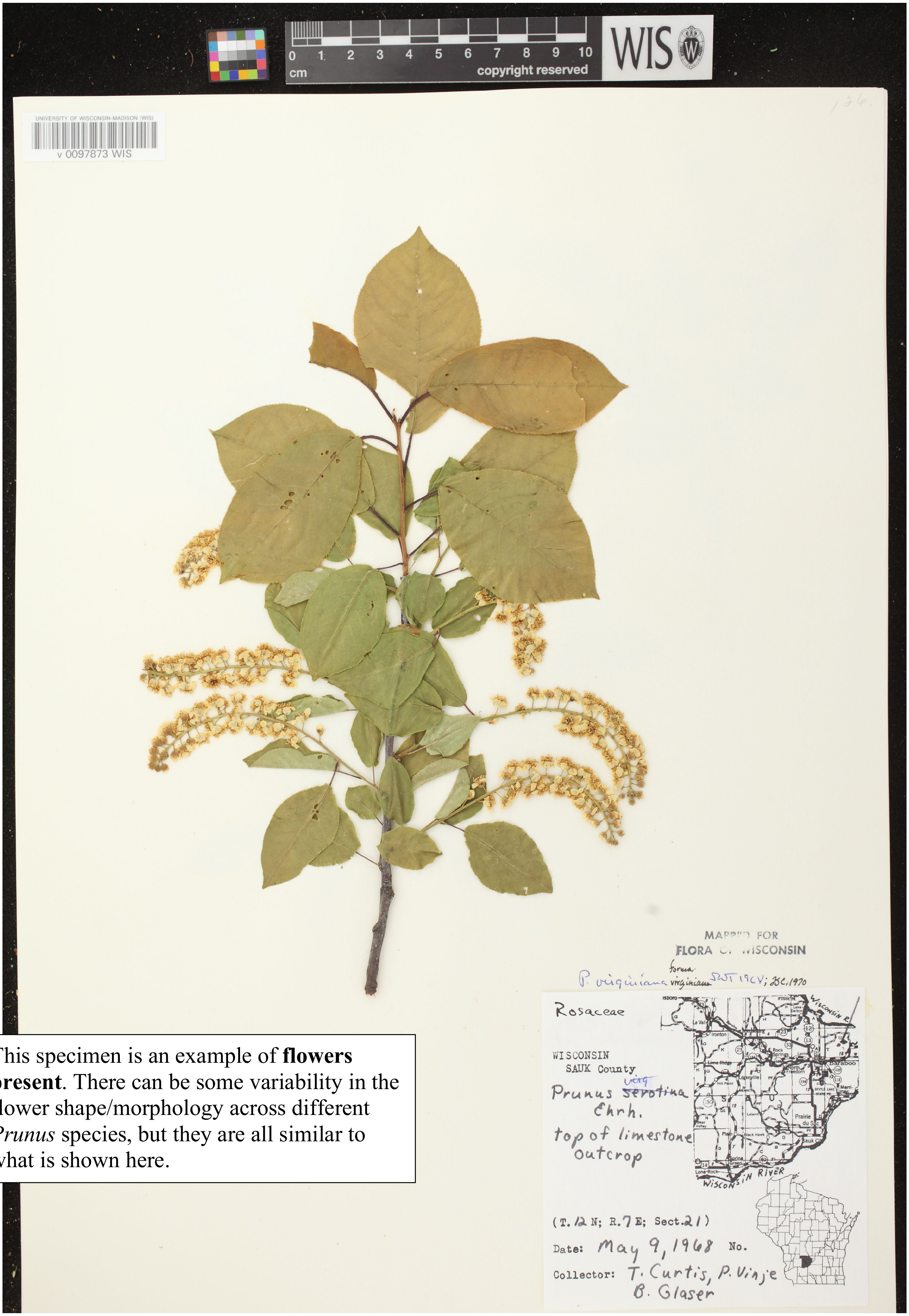

This specimen is an example of **flowers present**. There can be some variability in the flower shape/morphology across different *Prunus* species, but they are all similar to what is shown here.

DOI

This specimen is an example of **flowers present**. This specimen shows a different flower morphology, where the flowers are not on racemes, or stalk-like features, as on the first example.

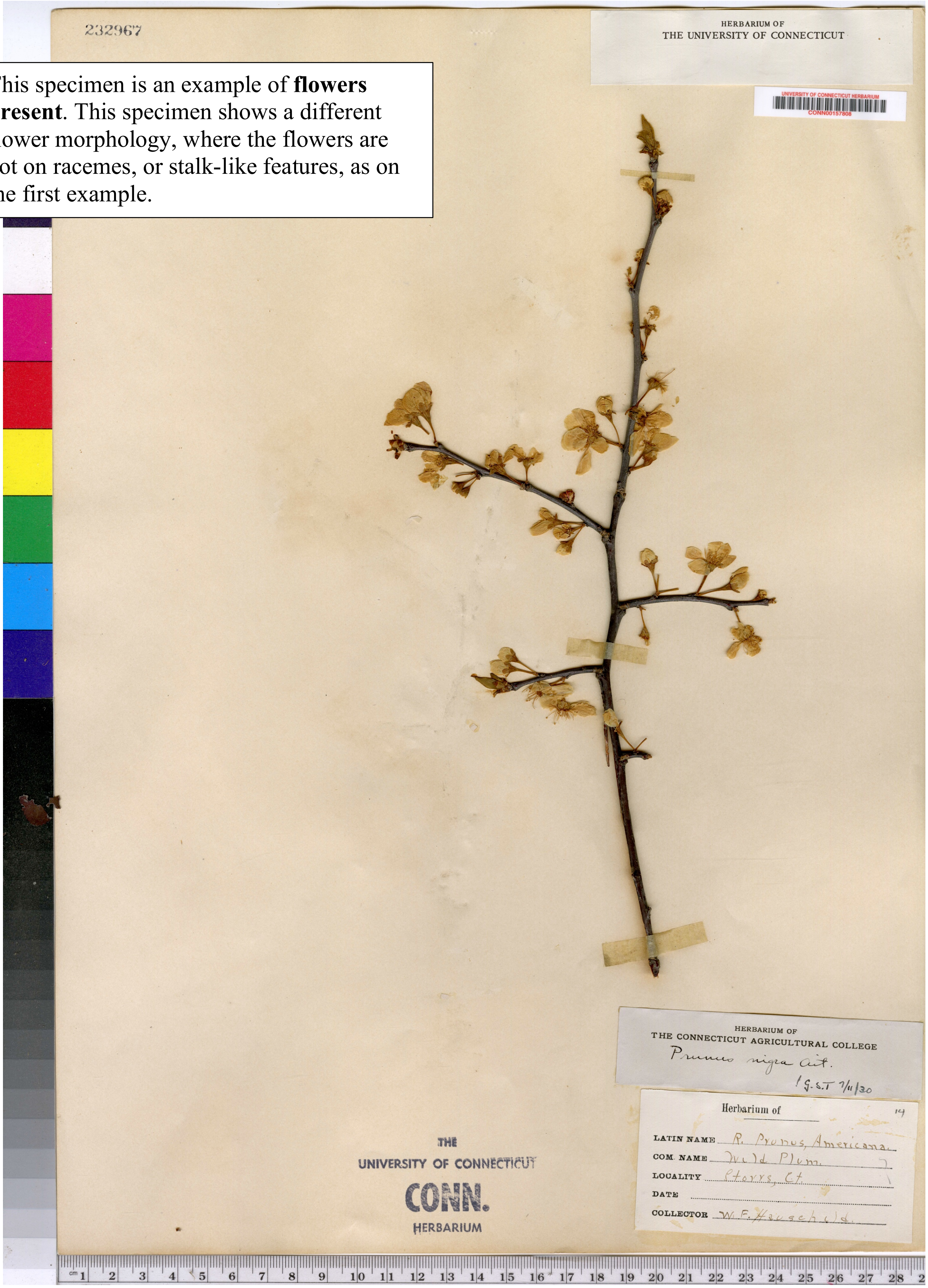

DOI

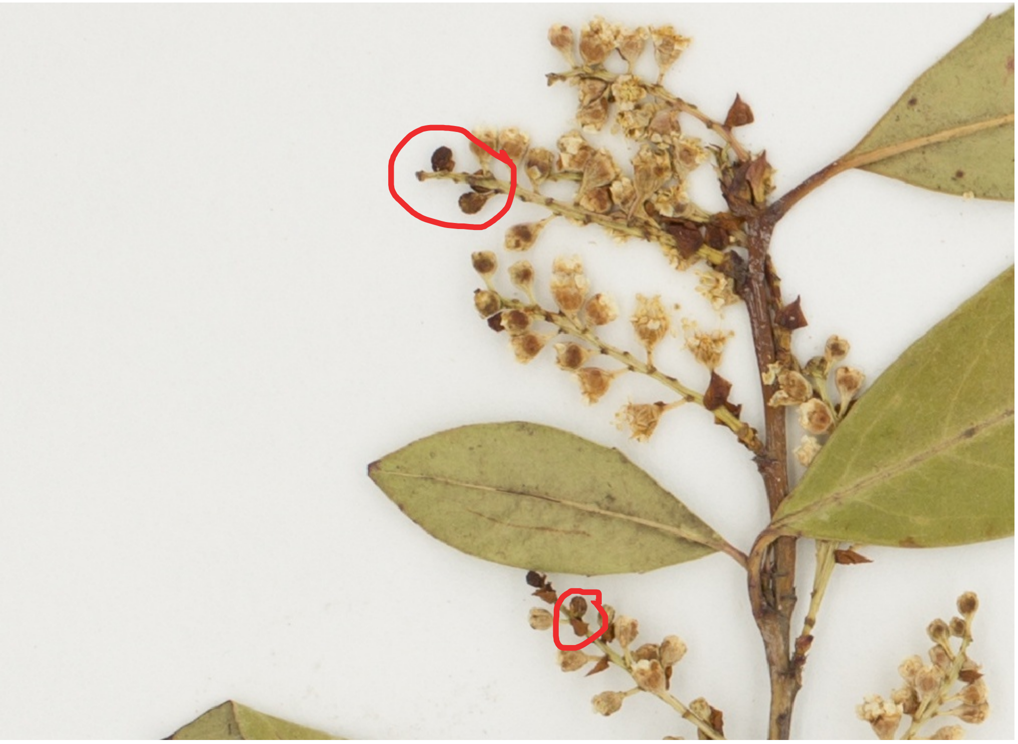

This image (above) is an example of **flowers present**. Sometimes flowers have not fully opened yet, and they can be seen as buds on specimens. There are some examples of buds (circled) and opened flowers on this image.

DOI

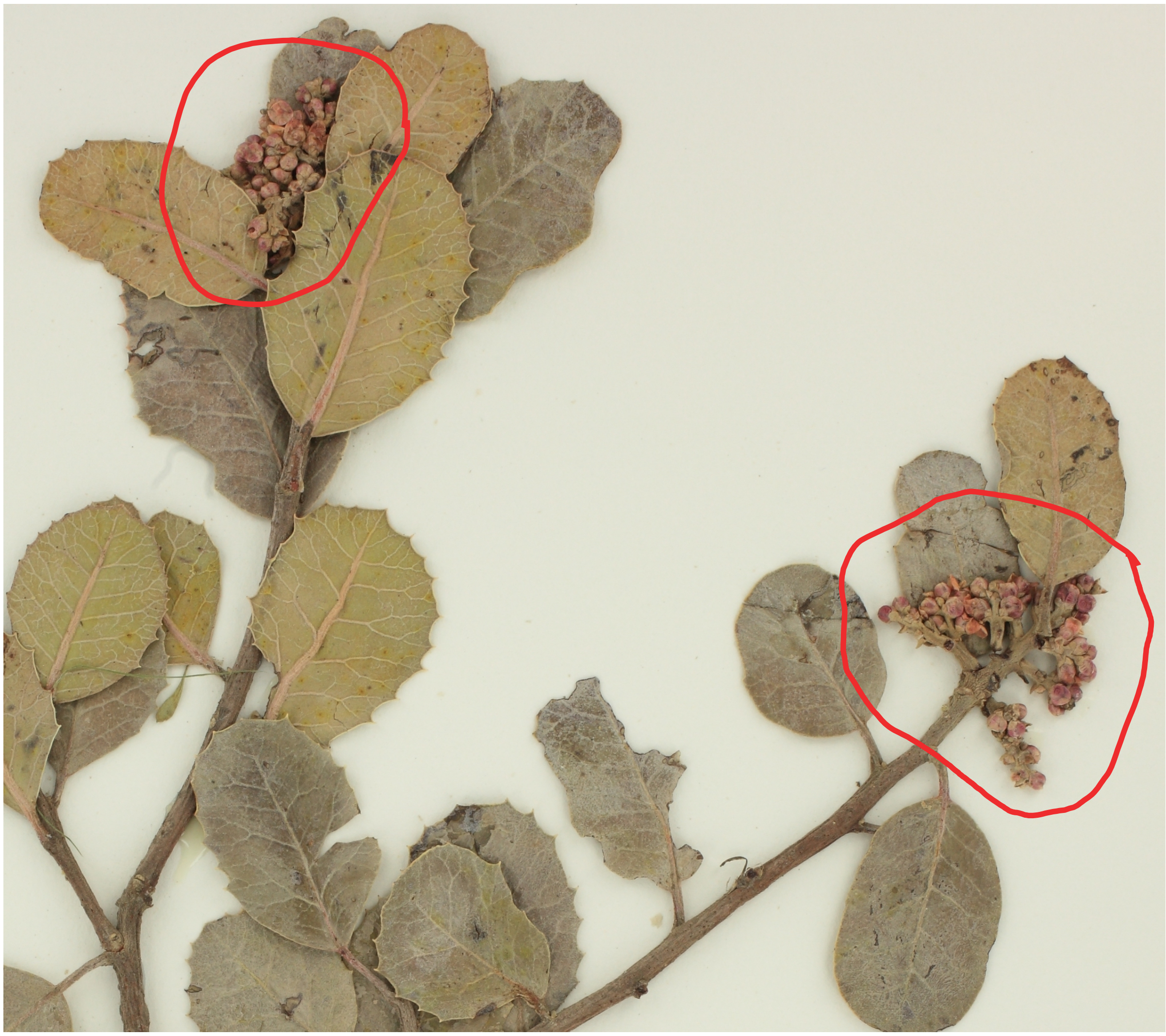

This image (above) is another example of **flowers present**. There are no opened flowers present on this specimen, but there are flower buds (circled).

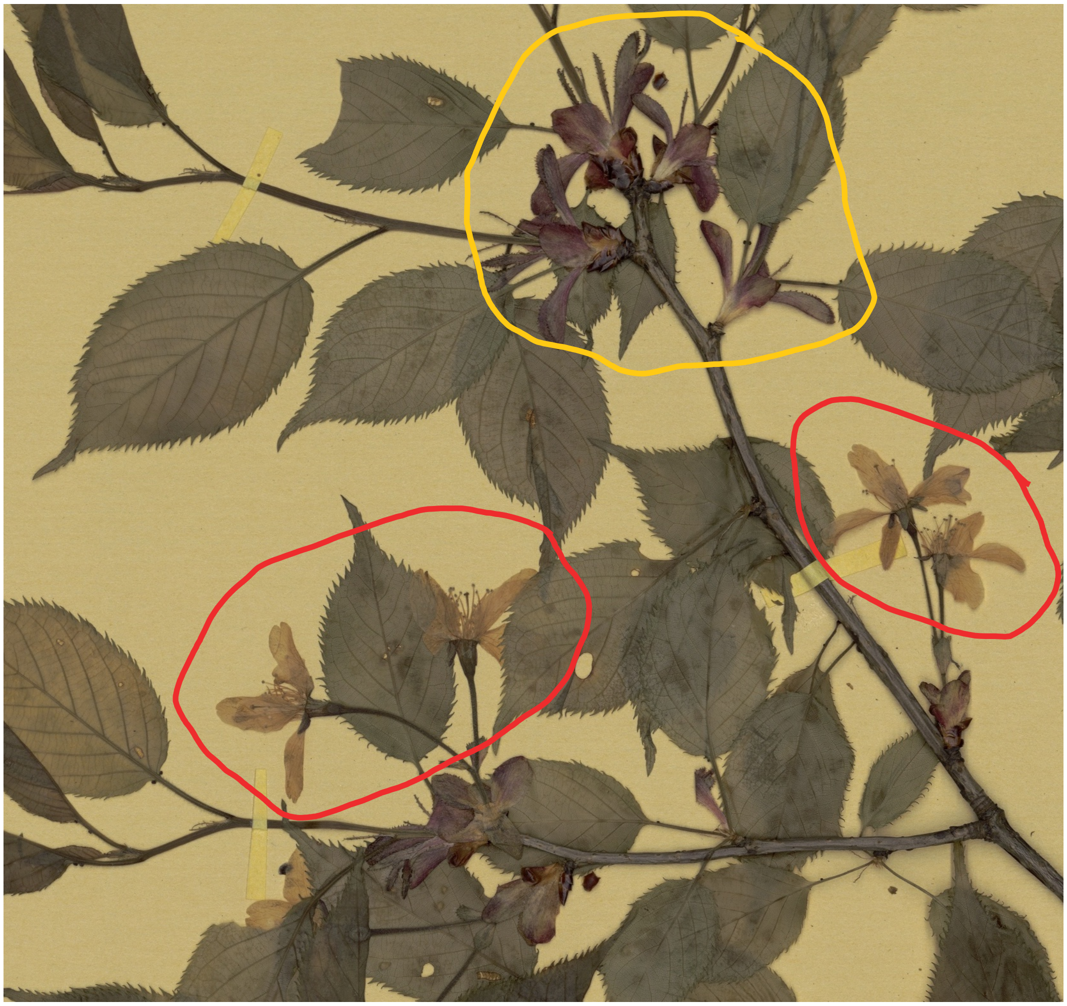

This image (above) is another example of **flowers present** (circled in red). However, there are some structures that may appear flower-like, but they are not actually flowers (circled in yellow). These are actually stipules.

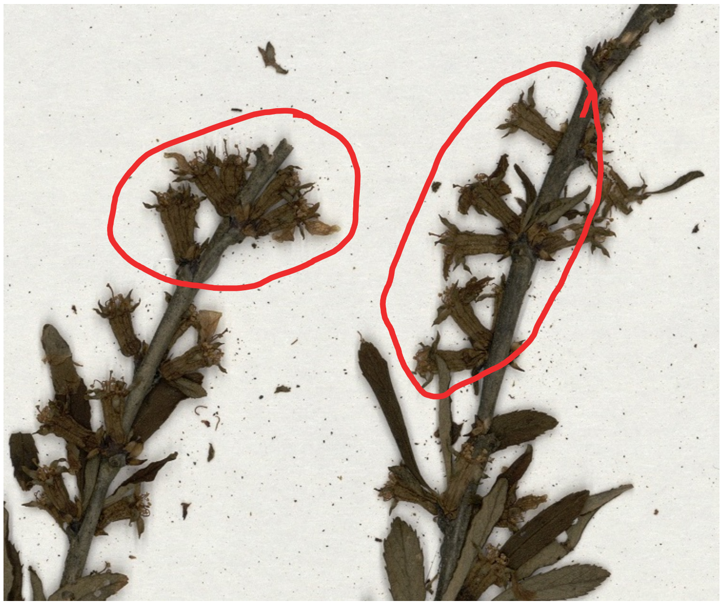

This image (above) is another example of **flowers present** (circled in red). Their morphology is slightly different than that of other *Prunus* species, but if you look closely, you can see the flower parts poking out of the tops of the flowers.

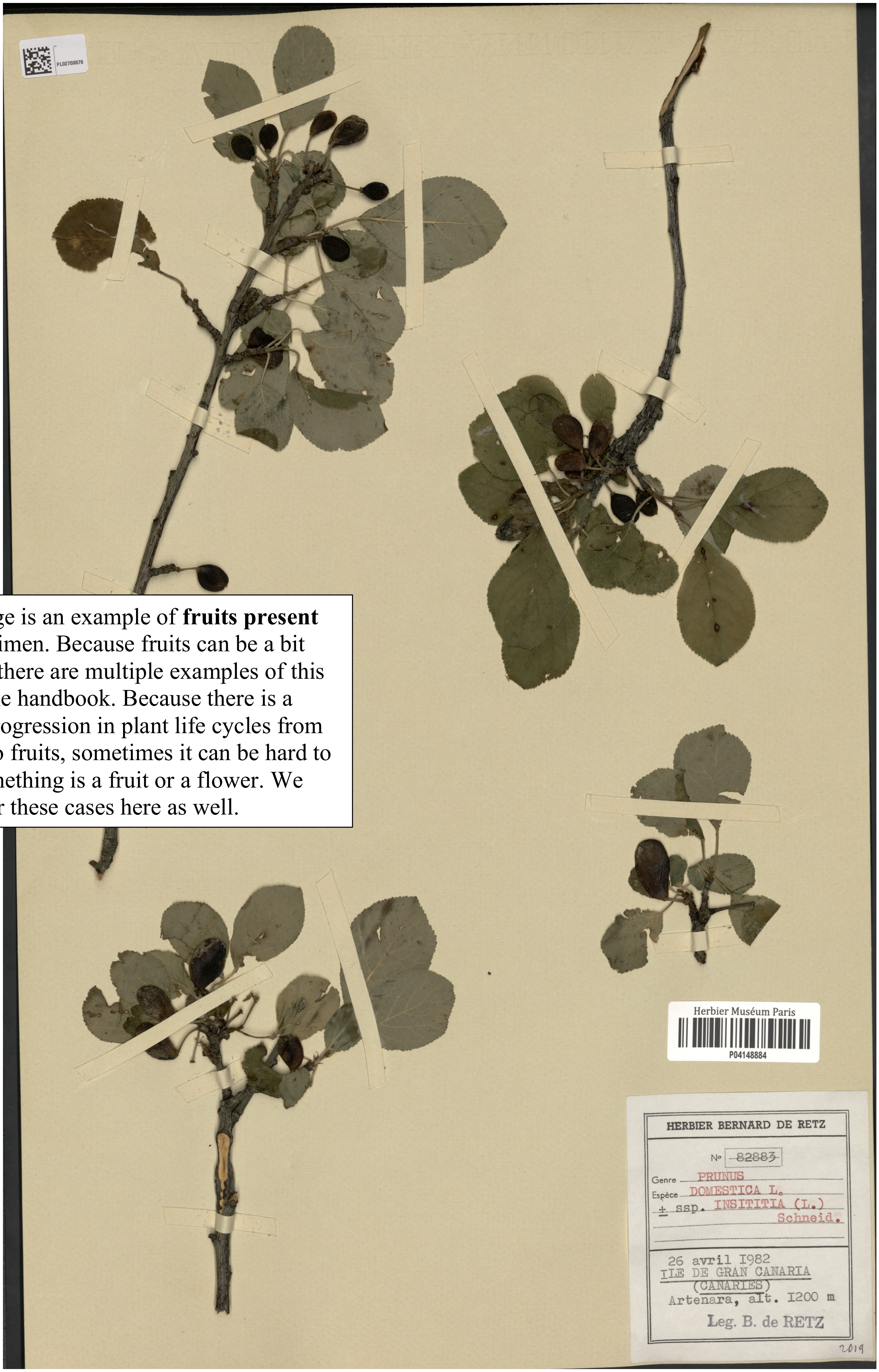

This image is an example of **fruits present** on a specimen. Because fruits can be a bit variable, there are multiple examples of this state in the handbook. Because there is a natural progression in plant life cycles from flowers to fruits, sometimes it can be hard to tell if something is a fruit or a flower. We will cover these cases here as well.

This image is an example of **fruits present** on a specimen. This example shows that sometimes fruits are not always just dark bulbs on *Prunus* specimens.

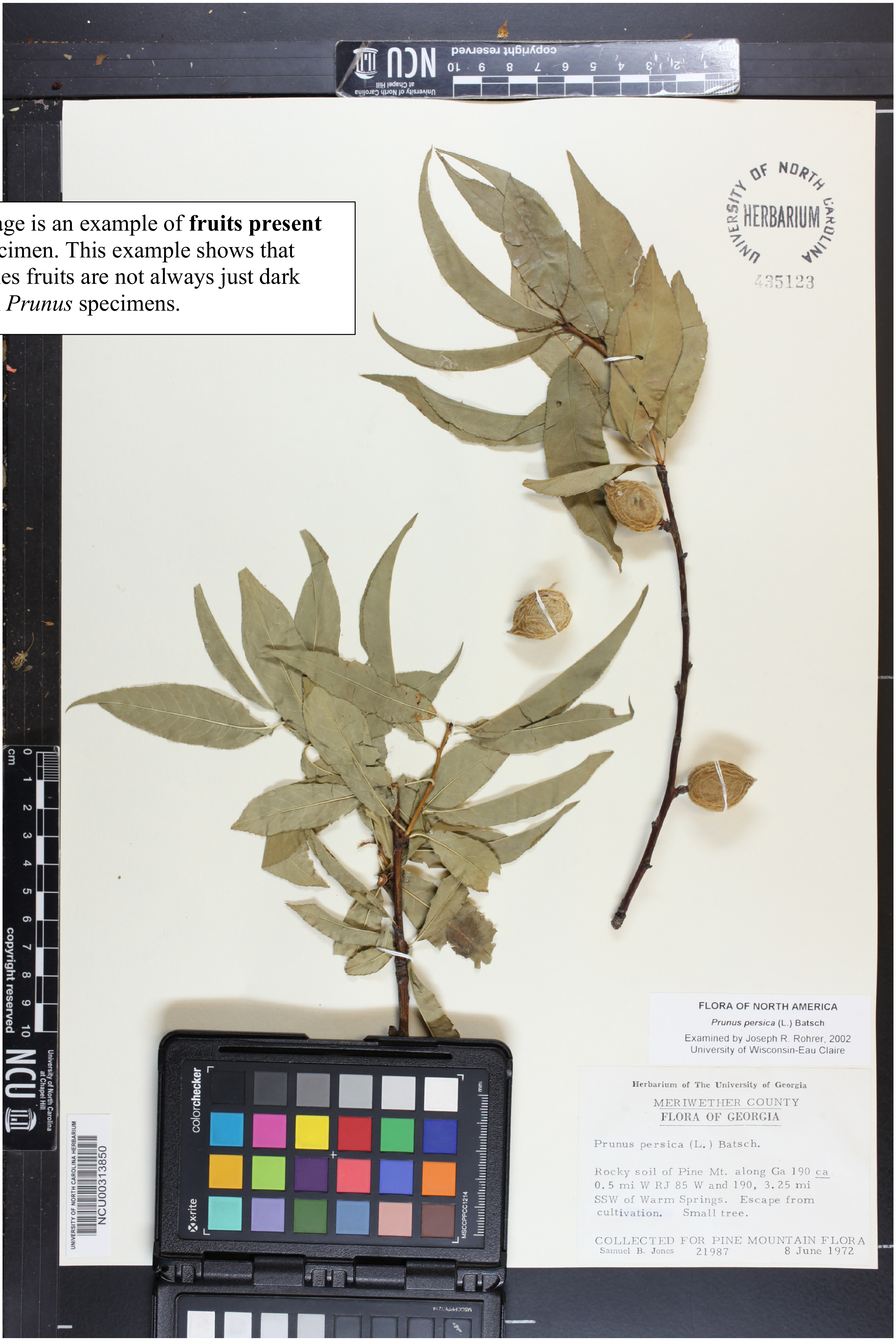

DOI

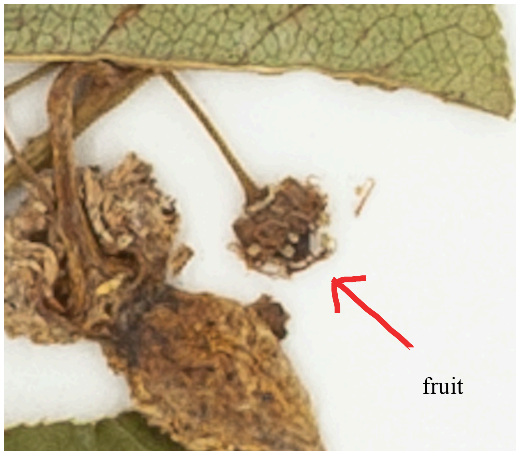

This example (left) shows a case where the specimen is borderline between flower and fruit. In cases like these, we score **fruits present** if there is enough fruit peeking out from the flower that it is visible.

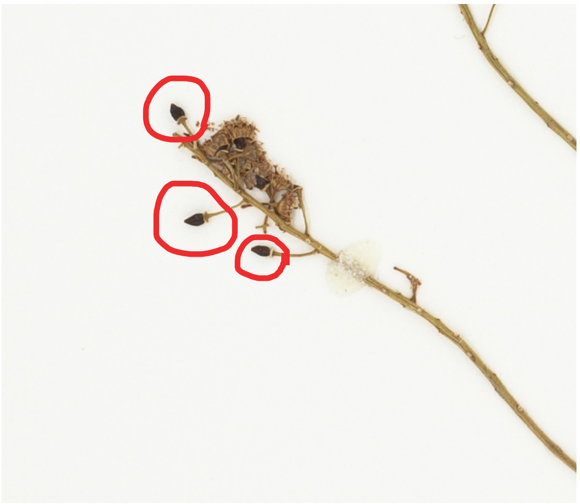

This example (left) shows a case where the specimen has immature fruits. We still score specimens like this as **fruits present**.

DOI

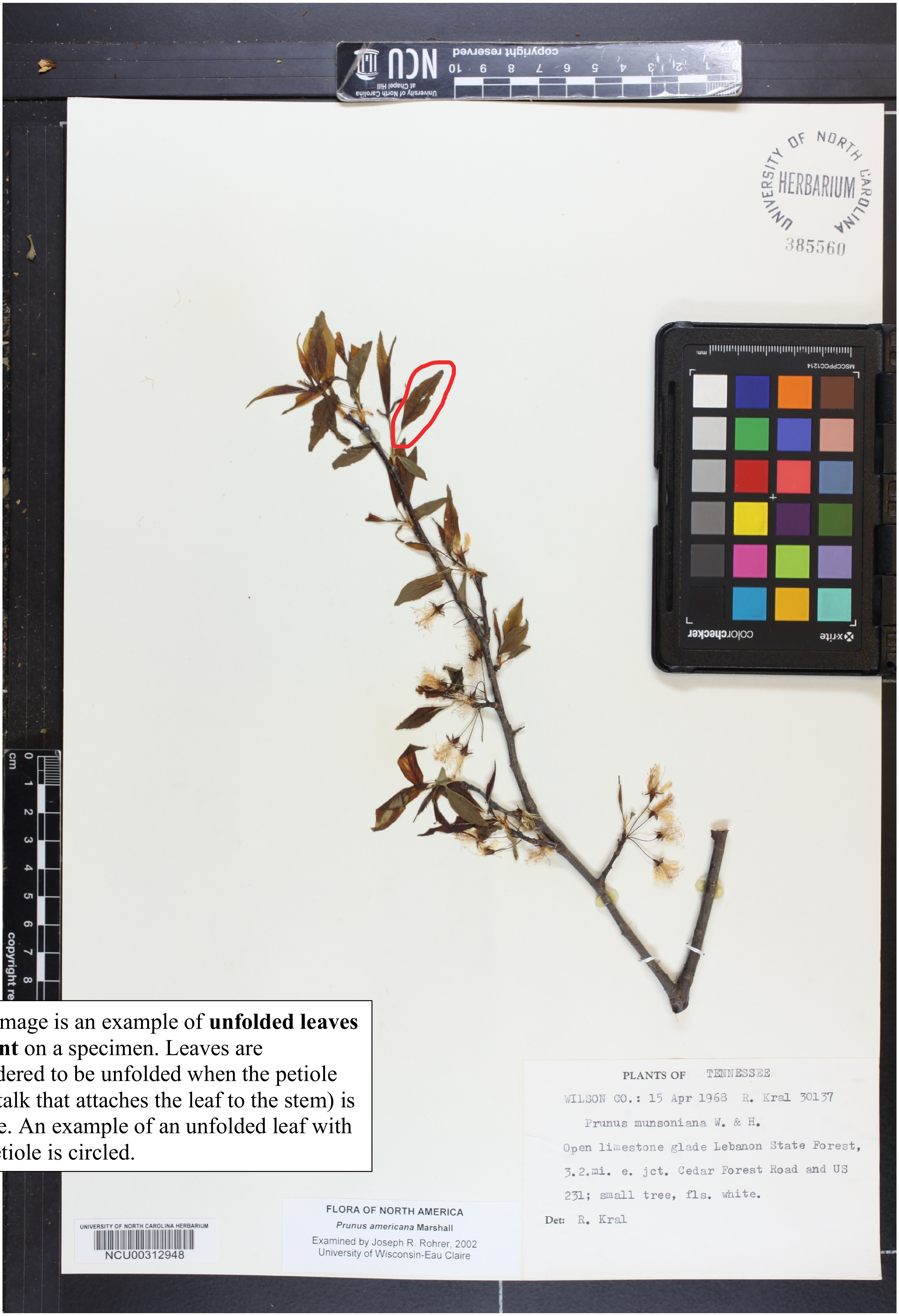

This image is an example of **unfolded leaves present** on a specimen. Leaves are considered to be unfolded when the petiole (the stalk that attaches the leaf to the stem) is visible. An example of an unfolded leaf with the petiole is circled.

This image is an example of **unfolded leaves ABSENT** on a specimen. Often times flowers and immature leaves on specimens can fool you into thinking there are unfolded leaves on a specimen, but look closely. There are no unfolded leaves with visible petioles on this specimen.

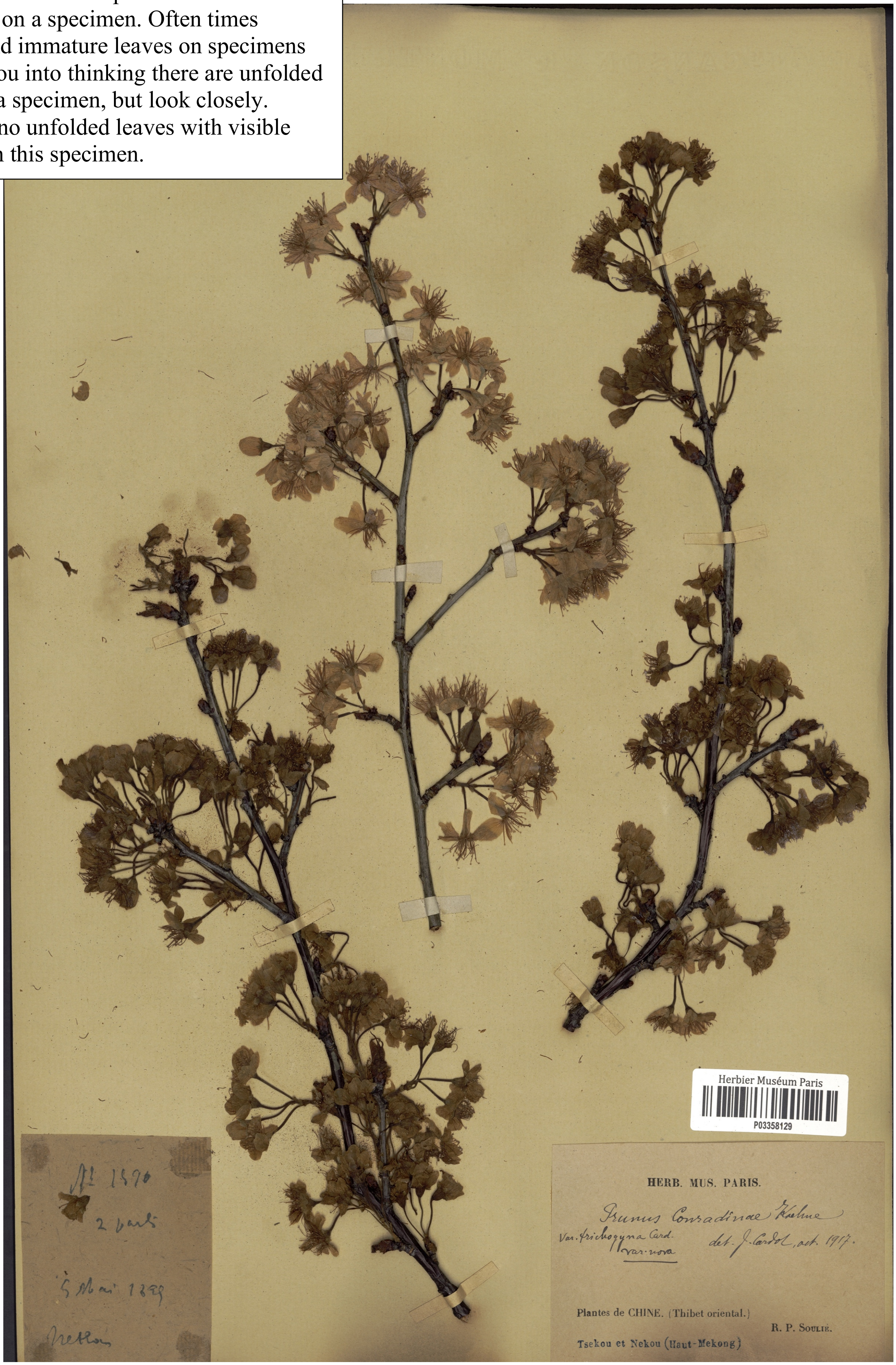

Supplement: Supplementary file 2 — APPENDIX S2. Herbarium scoring volunteer handbook for Prunus. [file APS3-8-e11370-s002.pdf]
