# Supplementary material for: Maximizing human effort for analyzing scientific images: A case study using digitized herbarium sheets
Source: Appl Plant Sci. 2020 Jul 1;8(6):e11370. doi: 10.1002/aps3.11370 (PMC7328657; doi:10.1002/aps3.11370)
Supplement: Supplementary file 3 — APPENDIX S3. Herbarium specimen machine learning survey. [file APS3-8-e11370-s003.pdf]

doi:10.1002/aps3.11370

Herbarium Specimen Machine Learning Survey

1. **Which of the following categories best describes your status? Circle one.**

    a. **Undergraduate student**
    b. **Graduate student**
    c. **Postdoctoral associate**
    d. **Faculty**

2. **Do you have any prior experience with botany research or identifying plant species? Circle one.**

    Yes **No**

Internal use ONLY → Identifier: ____________________
